# State-of-the-Art of (Bio)Chemical Sensor Developments in Analytical Spanish Groups

**DOI:** 10.3390/s100402511

**Published:** 2010-03-24

**Authors:** María Reyes Plata, Ana María Contento, Angel Ríos

**Affiliations:** Department of Analytical Chemistry and Food Technology, Faculty of Chemistry, University of Castilla, La Mancha, 13004, Ciudad Real, Spain; E-Mails: MariaReyes.Plata@uclm.es (M.R.P.); Ana.Contento@uclm.es (A.M.C.)

**Keywords:** (bio)chemical sensors, analytical chemistry, Spanish groups, state-of-the-art

## Abstract

(Bio)chemical sensors are one of the most exciting fields in analytical chemistry today. The development of these analytical devices simplifies and miniaturizes the whole analytical process. Although the initial expectation of the massive incorporation of sensors in routine analytical work has been truncated to some extent, in many other cases analytical methods based on sensor technology have solved important analytical problems. Many research groups are working in this field world-wide, reporting interesting results so far. Modestly, Spanish researchers have contributed to these recent developments. In this review, we summarize the more representative achievements carried out for these groups. They cover a wide variety of sensors, including optical, electrochemical, piezoelectric or electro-mechanical devices, used for laboratory or field analyses. The capabilities to be used in different applied areas are also critically discussed.

## 1. Introduction

The research and technology development of (bio)sensors has clearly increased in the last few years due to the necessity of solving current problems in various fields in our society. The conventional analytical techniques provide traceability, precision and accuracy, but in many cases they demand expensive and complex instrumentation, low analysis frequency, high reagent and sample consumption, lack of portability and need of skilled technicians [1,2]. For these reasons, there has been great interest in researching more selective chemical sensors towards a particular analysis, when sensors are applied to complex samples and sensitive devices to allow determination of lower concentrations, with low cost and easily handled instrumentation to perform *in situ* measurements [3].

A chemical sensor is formed by two integrated parts: a receptor element, which responds in a selective way, and a physical transducer element that converts the chemical information into an analytical signal. A biosensor contains an immobilized biological sensing element as a receptor element, which can bind with target analytes. (Bio)sensors are usually categorized according to the transducer type (e.g., electrochemical, optical, piezoelectrical or thermal), or the biorecognition principle (e.g., enzymatic, immunoaffinity recognition, whole-cell sensor or DNA) [4,5]. Thus, (bio)sensors are devices that recognize and quantify specific molecules. The major areas of applications are in environmental monitoring, medical and health diagnosis, industrial safety, security for military applications, surveillance, and the automotive industry.

(Bio)sensors can be defined as an ideal and useful tool to carry out real-time analysis simply. The analyte is physisorbed or chemisorbed onto the sensor in a reversible or irreversible process, which induces a response [6]. In this respect, research activities in (bio)sensors must be focused to get reliable, accurate, portable, sensitive, and fast sensors, due to their ability to operate at lower-power, small-size and relatively low cost. This objective is very ambitious and, for this reason, an interdisciplinary endeavor is clearly important to achieve it. Towards this goal, physicists, engineers, chemists and biologists are sharing their knowledge, tools, techniques and information to develop hardware and modify sensor surfaces from a chemical and biological viewpoint.

In the chemistry field, the term (bio)chemical sensor is more widely used in analytical chemistry. The major area of interest today in the analytical sensor field is the use of new materials with molecular recognition properties to carry out direct measurements without the necessity for a previous separation step [7]. Recently, certain nanomaterials are attractive candidates because of their small size (1–100 nm) and, correspondingly, large surface-to-volume ratio, chemically tailorable physical properties, which directly relate to size, composition and shape, unusual target binding properties and overall structural robustness [8]. Nanomaterials such as nanoparticles or carbon nanotubes connected with biomolecules are being used for several bioanalytical applications [9]. MIPs, organic dyes and metal complexes have led sensor modifications to improve selectivity, a marked sensitivity and simplification of the analytical devices.

The constant improvements in microfabrication techniques, and the rapid development of new nanofabrication techniques, have allowed the production of functional micro and nanoscale structures and devices, and therefore, the development of micro total analytical systems (μTASs) with the additional advantage of miniaturization [10]. All features found in traditional analytical systems must also be provided in small portable instrument based on miniaturized disposable cartridge systems incorporating either electrochemical or optical chemo/biosensing [7]. The new generation of these chemical analyzers (μTASs) have induced the integration of scaling down of all the unitary operation of the analytical process [2]. Specifically, the miniaturization of analytical systems has been developed in the fluidic field due to the development of microfabrication of microdevices, such as micropumps, microreservoirs, microchannels and micro filters [2], but the development of new materials has allowed a new objective in the research of micro and nanosensors. Therefore, trends in the sensor field address the possible combination of bulk sensor with microsystems. The goal is to link the advantages obtained by the telecommunication and microelectronics technologies [11].

**Figure 1.**
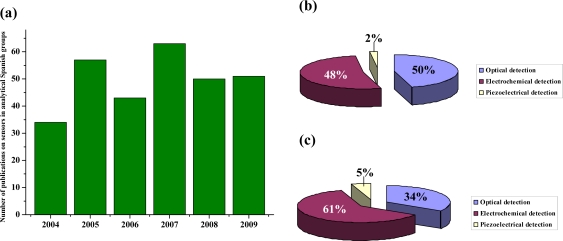
(a) Number of publications on sensors from analytical Spanish groups during 2004–2009. Distribution of publications according to the type of transduction used in Spanish (b) and international (c) analytical groups.

This review presents the state-of-art of the sensor field, from an analytical point of view, from Spanish groups. It covers the current state of modes of detection, design considerations and innovative applications on sensors. The review is focused on the time period from 2004–2009. Data were electronically searched in SciFinder and Web of Knowledge databases. The number of publications during this period is represented in Figure 1a, demonstrating a great interest in the development of sensors by analytical Spanish groups (listed in Table 1). These publications summarize the latest advances and future trends in producing, modifying, characterizing and integrating sensors. Figure 1b shows a statistical study of the different transduction techniques used by Spanish groups, and it can be compared with those in the worldwide analytical field (Figure 1c). As it can be seen, the primary detection technique used by analytical Spanish groups has been optical detection (50%), followed by electrochemical detection (48%), and piezoelectric detection only accounts for 2%. However, in the international analytical arena, the primary technique of transduction is electrochemical detection (61%), followed by optical detection (34%), and finally, piezoelectric detection (5%). In the first part of the review, the use of different materials and technologies in chemical and biochemical sensors is reported. The capabilities and applications are discussed. Nanoparticles (NPs), carbon nanotubes (CNTs), quantum dots (QDs), magnetic beads, metal nanoclusters, sensor nanofilms, as well as molecular imprinted polymers (MIPs), metal complexes sol-gel materials, organic ligands and other materials are discussed. Finally, the commercial sensors produced by analytical research groups are shown.

## 2. Materials and Technologies in Chemical and Biochemical Sensors

### 2.1. Nanomaterials and nanotechnologies

Nanotechnology implies manipulating individual atoms, molecules or nanosized objects with the aim to develop materials with novel properties and behavior that are not displayed by the bulk matter with the same condition. Nanoscale science involves substances on the nanometer scale: at least one dimension in the order of less than 100 nm. Nanomaterials and nanotechnologies are extensively applied in sensor designing [12,13]. Rius *et al.* introduced and discussed the main concepts behind the development of nanosensors and the most relevant applications in the field of environmental analysis. They discussed several nanostructures that are currently used in the development of nanosensors [13]. A brief summary of the main nanomaterials that are currently used in the development of nanosensors was presented.

#### 2.1.1. Carbon nanotubes

Carbon nanotubes (CNTs) have generated great interest based on their field emission and electronic transport properties [14], their high mechanical strength [15] and their chemical properties. Originally, nanotubes were applied mainly in field effect transistor (FET)-based sensors, but since recently they have also been used in electrochemical sensors improving the capture of signal [12]. The group of F.X. Rius has reported several sensors based on a field effect transistor (FET) in which a network of single-walled carbon nanotubes (SWCNTs) acts as the conductor channel. Biosensors with estrogen receptor alpha (ER-α) acts as the sensing part to recognize bisphenol A in water [16], monoclonal anti-Candida antibodies provide specific binding sites for fungal antigens (*Candida albicans*) [17] and human immunoglobulin G antibodies reacts with human immunoglobulin G [18,19].The group of S. Alegret and A. Merkoçi have developed several electrochemical sensors using CNTs. A novel application of multi-walled carbon nanotubes (MWCNTs) for biosensor was presented. *β*-Cyclodextrin (*β*-CD) as a molecular receptor and MWCNTs as an enhancer of electron transfer are integrated in a dopamine (DA) electrochemical sensor system. The proposed molecular host–guest recognition based sensor had a high electrochemical sensitivity for the determination of DA [20]. The nanotubes can be incorporated into a polymer coating deposited onto the electrode surface [12]. In this respect, a new report of A. Merkoçi’s group was presented. A carbon paste electrode (CPE) using supramolecular systems, such as MWCNTs, *β*-cyclodextrin (*β*-CD) and a new conducting polymer was electrochemically formed on the CPE, via polymerization of the *β*-CD, and determination of dopamine and ascorbic acid were carried out [21]. Surface modification of carbon nanotubes with polymers films enhances the selectivity of sensors. Pingarrón and co-workers have constructed a biosensor based on the immobilization of the enzyme lactate dehydrogenase (LDH) on a glassy carbon electrode modified with a hybrid carbon nanotube-conducting polymer (poly (3-methylthiophene)). The biosensor has good results determining lactate when the biosensor is coated with a Nafion film [22]. Merkoçi and Alegret have constructed rigid and conductive carbon nanotube-epoxy composite (CNTEC) electrodes and the behavior of the electrodes was characterized by using cyclic voltammetry of ferricyanide, NADH and hydrogen peroxide [23]. They reported a novel glucose biosensor based on the immobilization of glucose oxidase (GOx) on a rigid and renewable CNT epoxy-composite matrix prepared by dispersion of MWCNTs inside the epoxy resin. The use of CNT as the conductive part of the composite ensures better incorporation of enzyme into the epoxy matrix and faster electron transfer rates between the enzyme and the transducer [24]. The report includes the development of a microbial biosensor based on a carbon-nanotube epoxy composite (CNTEC) platform used as supporting electrode for cell immobilization. For this purpose, *Pseudomonas fluorescens* cells were immobilized on the surface of the CNTEC electrode by means of gelatin which it was then cross linked with glutaraldehyde [25]. Merkoçi *et al.* have presented a biosensor based on tyrosinase-integrated (Tyr) CNT epoxy composite electrode (CNTECE) to perform the determination of catechol. The modified electrode was electrochemically characterized by amperometric and voltammetric techniques [26]. A novel approach for the fabrication of polymer–CNT based biosensors was presented by Lechuga and co-workers. The polymer–CNT composite was drop cast on the top of microfabricated electrodes, resulting in a huge increase in the electrochemical area, opening up the possibility of binding biomolecules to the MWCNT wall. The resulting composite surface appears to be covered by a polymer layer surrounding the CNT, which is partially removed by a plasma treatment. The process is fully compatible with microelectronic fabrication technology ,and therefore the devices can be batch processed. Covalent immobilization of appropriate immunochemical receptors to the surface of CNTs enables the development of immunosensor platforms. Their performance is tested with rabbit immunoglobulin G (IgG), chosen as the model analyte [27]. The obtained results demonstrated remarkable electrochemical and mechanical advantages of carbon nanotube composites compared to graphite composites for sensor applications.

#### 2.1.2. Noble metal nanoparticles

Metal nanoparticles (MNPs) research is an interesting field because of the unique physical and chemical properties (e.g., electrical, magnetic, optical, ionization potentials, *etc.*), which are distinct from those of both bulk metals and isolated atoms and molecules. However, MNPs have a great trend for aggregation, and for this reason can be unstable and lose their special properties. Moreover, MNPs represent an excellent biocompatibility with biomolecules and display unique structural, electronic, magnetic, optical and catalytic properties which have made them a very attractive material [28] as labels in the detection of DNA hybridization [28] using optical methods or various electrochemical techniques among other applications [30]. MNPs have been known since antiquity and have been used in optical and electrochemical sensors. Gold nanoparticles (AuNPs) are the most frequently used metal NPs in bioanalysis. Spanish research groups have only used electrochemical detection with MNPs, which is now summarized. Domínguez *et al.* reported a chemically derivatized horseradish peroxidase on biomimetic silica AuNPs for amperometric sensing applications. Scanning electron microscopy shows evidence of the formation of enzyme-modified nanospheres using poly(ethylenimine) as a template for silicic acid condensation. The modified nanoparticles were directly deposited on graphite electrodes. The *in situ* biomimetically synthesized peroxidase nanospheres are catalytically active, enabling direct bioelectrocatalysis at 0 mV *versus* Ag|AgCl with long-term stability [31]. In a new report, novel nanoelectrode arrays with enhanced electrochemical properties were developed as a general platform for electrochemical biosensors with the enhanced current outputs controlled by the structure of the self-assembled nanowires. The conducting nanowires were formed upon self-assembly of Au-shell/CoFe_2_O_4_-magnetic core nanoparticle on an Au electrode surface, which caused an increase of the electrode surface area yielding an electrochemical response to a diffusional redox probe. The primary electrochemical reaction of the electron relay was coupled with the bioelectrocatalytic oxidation of glucose in the presence of soluble glucose oxidase resulting in the amplification of the biocatalytic cascade controlled by the growth of the nanostructured assembly on the electrode surface. The process was characterized by *in situ* electrochemical measurements showing the enhanced electrochemical signals upon generation of the nanostructured interface [32]. The analysis of specific gene sequences in the diagnostic laboratory is usually based on DNA hybridization, in which the target gene sequence is identified by a DNA probe able to form a double stranded hybrid with its complementary nucleic acid with high efficiency and specificity [33]. MNPs, in general, and particularly AuNPs offer attractive properties to act as DNA hybridization tags with interest in developing sensitive electrochemical genosensors [34]. The most important strategies used to integrate AuNPs in DNA detection systems are: (a) the electrochemical detection of AuNP label by detecting the gold ions released after acidic dissolving; (b) direct detection of AuNPs anchored onto the surface of a conventional genosensor (based on stripping voltammetry); (c) silver enhancement using conductometric technique; (d) enhancement of AuNPs anchored to conventional genosensor surface by using silver or gold; (e) using AuNPs as carriers for other electroactive labels. Direct detection of AuNPs, but not in connection with the detection of DNA hybridization, was earlier reported by the groups of Alegret and Costa-García [35,36]. The application of AuNPs as oligonucleotide labels in DNA hybridization detection assays using a magnetic graphite-epoxy composite electrode (M-GECE) has been reported by Alegret *et al.* [37]. The novel gold nanoparticle-based protocol for detection of DNA hydridization was based on a magnetically trigged direct electrochemical detection of gold quantum dot tracers. Merkoçi and Alegret [38] reported two AuNPs based genosensors designs for detection of DNA hybridization. Both assay formats were also based on a magnetically induced direct electrochemical detection of the AuNPs tags on M-GECE. Lorenzo *et al.* have presented new genosensors modifying gold electrodes with metal complexes. In this way, the authors have been addressed genosensors based on ruthenium complex generated *in situ* [39] and based on gold nanoparticles (Au-NPs) in conjunction with an “*in situ*” prepared ruthenium complex as sensitive and selective electrochemical indicator in gold electrodes [40,41]. The gold electrodes were modified with DNA and pentaamin ruthenium [3-(2-phenanthren-9-yl-vinyl)-pyridine] [39]. A new sensor implies a recognition surface based on nanoparticles modified with a thiolated capture strand able to hybridize it complementary sequence and the hybridization event is detected using a water-soluble pentaamin ruthenium [3-(2-phenanthren-9-yl-vinyl)-pyridine] complex. Emphasis has also been placed on the synthesis of chemical materials in order to be used as new electrochemical indicators in DNA sensing, such as transition metal complexes and Schiff base ligands [40]. In this case, both the hybridization event and electrochemical detection take place on the same surface [41]. The genosensors have been applied to detect complementary target sequences of *H. pylori* with electrochemical detection. AuNPs have been employed for easily completing direct electron transfer from redox protein to the electrode because their electronic conductivity, chemical stability and biocompatibility are better than those of inorganic NPs. Electrochemical deposition of AuNPs on a carbon-paste electrode has been reported by the group of J.M. Pingarrón, as a method for creating a more favorable AuNP-modified biosensing interface [42]. Compared with the traditional process, this method is simpler and quicker, the performance condition is more moderated and it is suitable for selective deposition of thin film with controllable thickness. M.J. Arcos Martínez’s group has described several carbon screen-printed electrodes (SPCE) modified by direct electrochemical deposition with MNPs as AuNPs, silver nanoparticles (AgNPs) and platinum nanoparticles (PtNPs). AgNPs sensor has been applied to determine Sb (III) in seawater samples and pharmaceutical preparations [43], and lamotrigine in pharmaceutical preparation using pulse anodic stripping voltammetry [44]; whereas AuNPs sensor has been used in Sb determinations [45] and the PtNPs sensor has been designed to determine arsenic (III) [46]. On the other hand, AgNPs and AuNPs sensors have been applied to determine chromium (VI) by differential pulse voltammetry (DPV) [47]. Moreover, SPCEs and AuNPs/SPCEs were used as supports for the crosslinking immobilization of the enzyme urease for measurement of Hg (II), based on the inhibitive action of this metal on urease enzyme activity. The enzymic amperometric procedure was applied to determine Hg (II) levels in spiked human plasma samples [48]. In addition, this group has developed carbon and gold screen printed electrodes (SPCEs and gold SPEs) based on an easy covalent immobilization of the enzyme. The linkage of biomolecules through 4-nitrobenzenediazonium tetrafluoroborate, mercaptopropionic acid and thioctic acid monolayers has been attempted using bare SPCEs and gold SPEs, as well as, gold nanoparticles (AuNPs) modified SPCEs and gold SPEs. Direct covalent attachment of cytochrome P 450 2B4 (CYP450 2B4) to the transducer was carried out by carbodiimide and hydroxysuccinimide. The determination of phenobarbital (PB) was performed by this methodology [49].

Alegret *et al.* reported the development of novel approaches for inter-matrix synthesis and characterization of polymer stabilized metal nanoparticle (PSMNP) to resolve the stability problem. The synthesis of platinum and palladium polymer-stabilized metal nanoparticles (PSMNP) was carried out for the first time. PSMNP can be applied in molecular recognition devices in modified graphite-epoxy composite electrodes such as, sensors and biosensors [50,51]. Another solution of this problem is the use of core-shell PSMNP, which are composed of a cheap metal core coated with a thin noble metal shell [52–55]. The presence of both PSMNP along with an enzyme inside the polymeric membrane, which serves as a sensing element in amperometric biosensors, can substantially enhanced the electron-transfer from the enzymatic reaction site to the electrode surface. In this paper, Alegret reported, for the first time, a simple method for the intermatrix system (IMS) and characterization of core–shell MNPs with core-copper coated with platinum shell (Pt@Cu-PSMNP) by using sulfonated poly(ether ether ketone) (SPEEK) membranes as a nanoreactor. The results on evaluation of performance PSMNP-based amperometric sensors prepared by deposition of MNP-containing membranes on the surface of graphite-epoxy composite electrodes (GECE) have demonstrated their applicability for quantitative detection of hydrogen peroxide [56–58].

#### 2.1.3. Quantum dots

Quantum dots (QD) are colloidal nanocrystalline semiconductors, roughly spherical, with particle diameters typically ranging from 1–12 nm [49,60]. At such small sizes, these nanostructured materials behave differently from bulk solids, because of quantum-confinement effects [60]. In fact, when synthesized at the nanometer size and after adequate surface protection, these compounds develop intense and long-lasting luminescent emission with very narrow emission bandwidths (full width at half-maximum of approximately 15–40 nm). QDs typically have higher fluorescence quantum yields and better chemical and photoluminescence stability than conventional organic fluorophores. Furthermore, these nanocrystals have size-dependent tunable photoluminescense emission. The frequency of the light emitted by a specific quantum dot is related directly to its size; smaller particles tend to emit higher-energy (shorter wavelength) radiation. QDs also have unique attributes that make them superior to commercially available organic dyes when used for optical sensing. A. Sanz-Medel *et al.* review progress in exploiting the attractive luminescent properties of QDs in designing novel probes for chemical and biochemical optical sensing [61]. The same authors have proposed methods based on measurement of the luminescence deactivation ratio of surface-modified water-soluble QD for optical monitoring of Cu(II) [62] and cyanide [63].

#### 2.1.4. Magnetic beads

Magnetic beads are a powerful and versatile tool in a variety of analytical and biotechnological applications [64]. The use of non-porous magnetic beads greatly improves the performance of the immunological reaction, due to an increase in the surface area, and the faster assay kinetics achieved because the beads are in suspension and the analytical target does not have to migrate very far. According to their properties and separation steps, the matrix effect is minimized, despite this increased surface area. Additionally, the magnetic beads can be easy manipulated by using permanent magnets or electromagnets. Therefore, the analysis of samples performed on magnetic beads can be easily achieved without any pre-treatment steps of the sample. Alegret *et al.* developed a serial of sensitive, selective and rapid genomagnetic assays, based on the enzymic labelling with electrochemical detection of DNA by novel magneto electrodes. The DNA target is selectively bound by its hybridization with the biotinylated capture probe on magnetic beads. This protocol is quite promising for numerous applications in different fields as clinical analysis, environmental control as well as other applications [40,65–67]. This group has reported two magneto-immunosensing for the detection of sulphonamide antibiotics in milk and folic acid in vitamin-fortified milk with electrochemical detection using magneto sensors. The immunological reaction for the detection of sulfonamide antibiotics performed on the magnetic bead is based on a direct competitive assay using a tracer with HRP peroxidase for the enzymatic labeling, whereas in the case of folic acid the best performance was achieved with an indirect competitive immunoassay [68,69]. Merkoçi and co-workers have worked in a built-in magnet carbon electrode that allows the collection/immobilization on its surface of the microparamagnetic beads with the immunological sandwich and gold nanoparticle catalysts attached onto. The developed magnetoimmunosensing technology allows the antigen detection with an enhanced sensitivity due to the catalytic effect of gold nanoparticles on the electroreduction of silver ions. This method allows the obtaining of a novel immunosensor with low protein detection limits, with special interest for further applications in clinical analysis, food quality and safety as well as other industrial applications [70]. A mixture that contains glucose oxidase, amphiphilic pyrrole monomer and microbeads was deposited on a platinum electrode to prepare a glucose biosensor. The electrochemical polymerization of polypyrrole films onto magnetically immobilized hydrophilic microbeads was also carried out [71].

#### 2.1.5. Metal nanoclusters

Metal nanoclusters (MNC) are of great interest due to the special properties of nano-objects. In this case, the synthesis of polymer-stabilized metal nanoclusters of platinum (Pt-PSMNC) using the solid-phase-incorporated-reagents (SPHINER) technique was described. The synthesized MNC was used in the construction of new composite electrodes and offered advantages, such as a high electrical conductivity [72].

#### 2.1.6. Sensor nanofilms

Nanosized films include Langmuir-Blodgett films prepared by transfering monomolecular layers of organic molecules from a liquid subphase surface onto a solid substrate, and self-assembled structures like alkanethiol monolayers on gold surface [12].

##### Self-assembled monolayers

Self-assembly of monolayers (SAM) on the solid surface was discovered in the middle of the XX century. SAMs based on alkanethiols on gold surface are the most extensive application in chemical sensor designs and are resistant to water, as well as to acid and alkali solutions. They are extensively used for subsequent immobilization of biomolecules on the electrode surface [12]. Several modified gold sensors based on SAM have been developed by the group of J.M. Pingarrón to determine several analytes. An amperometric sensor based on mercaptopropionic acid (MPA) self-assembled monolayer (SAM) with horseradish peroxidase was immobilized by cross-linking with glutaraldehyde together with the mediator tetrathiafulvalene. The SAM-based biosensor was applied for the determination of hydrogen peroxide in rainwater and in hair dye [73]. Moreoever, several sensors to determine DNA have been fabricated. An interaction between the immobilized DNA and methylene blue (MB) was investigated using square wave voltammetry (SWV) to recognize double- or single-stranded DNA [74]. Additionally, *Escherichia coli* lac Z gene detection has been carried out using self-assembled monolayers immobilized in gold electrode [75]. The construction and performance of integrated amperometric gold electrodes biosensors for the determination of glycerol in wines [76], inulin in foods with gold nanoparticles [77] and fructose [78] have been evaluated. Different enzymes and compounds have been immobilized on a self-assembled monolayer (SAM)-modified gold electrode. The same group reported an amperometric immunosensor for the quantification of *Staphylococcus aureus* based on the coimmobilization of rabbit immunoglobulin G (RbIgG), tyrosinase on a mercaptopropionic acid self-assembled monolayer [79] and 3,3-dithiodipropionic acid di(N-succinimidyl ester)-modified gold electrode [80].

##### Langmuir-Blodgett films

Langmuir-Blodgett films have extensively used in electric sensors. Domínguez and co-workers have fabricated Langmuir-Blodgett (LB)-nanostructures as a nanoscale material for Ion selective Field Effect Transistors (ISFET). The electrochemical response of those monolayers modified with inophores onto ISFETs demonstrated the feasibility of this technology for sensor membrane fabrication and it opens further studies for other kind of devices such as optochemical sensors. This material modified with ionophores has applied in calcium and sodium determination [81].

### 2.2. Materials for chemical sensing

#### 2.2.1. Molecular imprinted polymers

Molecular imprinted polymers (MIPs), human-made polymers capable of recognizing a particular molecular in the presence of others due to the selective cavities of the material, have been successfully applied to the development of chromatography and solid phase extration methods and to the development of electrochemical, piezoelectric and optical sensors. In parallel with the classification of biosensors, MIP-based devices can work according to two different detection schemes: (1) affinity sensors (“plastic-bodies”) and, (2) catalytic sensors (“plastic-enzymes”) [82]. In this sense, Moreno-Bondi’s group have developed several MIPs to recognized penicillin-type β-lactam antibiotics (BLAs), [83,84] zearalenone (ZON) mycotoxin [85]. J.S. Durand Alegría *et al.* have synthesized several MIPs under different conditions using digoxin as template. The surface morphology was determined by scanning electron microscope (SEM) and the ability of the different polymers to selectively rebind the template was evaluated [86]. MIPs have been used to develop a competitive flow-through FIA assay for digoxin determination The MIP was packed into a flow cell and placed in a spectrofluorimeter to integrate the reaction and detection systems. The new fluorosensor showed high sensitivity and the selectivity was tested by determining the cross-reactivity of several compounds with structures analogous to digoxin. The optical sensor provided satisfactory results in analyses of this analyte in human serum [87]. Then, a comparative study of two automated flow-through fluorosensors for the determination of digoxin in serum samples has been carried out. An immunosensor with an anti-digoxin polyclonal antibody and a sensor with a selective reaction system based on a methacrylic molecularly imprinted polymer (MIP) synthesized by bulk polymerization was developed. No cross-reactivity with digoxin-related compounds was observed for this sensor at a digoxin/interferent ratio of 1:100. The lifetime of the immunosensor was about three months and the lifetime of the MIP sensor was over 18 months. Both sensors were used to determine the digoxin concentration of human serum samples with satisfactory results [88]. A new publication showed the synthesis and the performance of a molecularly imprinted polymer membrane for digoxin analysis [89]. The imprinted membrane was tested as the recognition element in a digoxin-sensitive fluorescence sensor. This simply manufactured MIP membrane showed good recognition characteristics, a high affinity for digoxin, and provided satisfactory results in analyses of this analyte in human serum. A novel molecular imprinted polymer (MIP) of high interest for room temperature phosphorescence (RTP) sensing systems is described by Sanz-Medel and co-workers. The synthesized MIP contains iodine as internal heavy atom in their polymeric structure. The synergic combination of a tailor-made MIP recognition with a selective RTP detection is a novel concept for optosensing devices which is assessed here for simple and highly selective determination of trace amounts of fluoranthene in water [90,91]. A. Fernández-Gutiérrez *et al.* developed a new MIP fluorescence opto-sensing flow injection system to determine monoamine naphthalene compounds, such as 1-naphthylamine (1-NA) and 2-naphthylamine (2-NA) in drinking water. The use of chemometrics tools, such as partial least-squares (PLS-1), multi-way PLS (N-PLS) and unfolded PLS (U-PLS) were satisfactory allowed by the simultaneous determination of principal monoamine naphthalene compounds, even in presence of the potential interferent, as 1-naphthalenemethylamine [92,93]. This group also proposed a simple and semi-empirical model to carry out the synthesis of homogeneous and transparent MIPs with volatile organic compounds (VOCs) in order to obtain optical sensing films. The novel polymers detect volatile organic compounds in water by measuring intrinsic fluorescence [94]. Tuñon Blanco *et al.* described a new methodology for the design of molecularly imprinted polymers (MIPs). The method allows the rational choice of the most suitable monomer and polymerization solvent among a set of chemicals traditionally used in MIP formulations for the molecular imprinting of a given template. It is based on the comparison of the stabilization energies of the prepolymerization adducts between the template and different functional monomers. A voltammetric sensor for homovanillic acid was constructed using different MIPs as recognition element, confirming that the solvent (toluene) and functional monomer (methacrylic acid) selected according to the theorical predictions lead to the most efficient molecular recognition sensing phase. The selectivity obtained for homovanillic acid over other structurally related compounds buttresses the validity of this strategy of design [95]. R.J. Barrio and co-workers synthesized several MIP-voltammetric microsensors based on a carbon fiber microelectrode (CFME). The polymeric synthesis was carried out by electrocopolymerization of aniline and o-phenylenediamine (o-PD) in presence of the template. The voltammetric microsensors was able to differentiate the DNOC [96] and metamitron [97] from other closely related compounds.

#### 2.2.2. Metal complexes

Fernández-Gutiérrez *et al.* designed several metal complexes with optical properties to prepare different sensors. The group developed iron(II) phthalocyanine complexes immobilized on nanostructured solid supports to prepare optical sensing layers and several studies such as optical properties, chemical variables, analytical features, selectivity rates, response times and type of nanostructure supports have been carried out [98]. A novel phosphorescent Ir (III) complex [Ir(2-phenylpyridine)2(4,4′-bis(2-(4-N,Nmethylhexylaminophenyl) ethyl)-2-2′-bipyridine)Cl] has been designed and synthesized, to be used as an oxygen probe. The complex was incorporated in a polystyrene and nanostructured metal oxide support and it was characterized by spectroscopic and analytical methods. The sensing film shows long-term stability (up to 12 months), complete reversibility of the signal quenched by oxygen and a quick response time to various oxygen concentrations [99]. A novel new gold-silver complex based on 2,2′-bipyridine, whose formula is {Au_2_Ag_2_(C_6_F_5_) 4[(C_5_H_4_N)-(C_5_H_4_N)]_2_}_n,_ was fabricated as fiber optic sensor. This material was used to detect VOCs such as ethanol, methanol and acetic acid [100]. M.E. Díaz García *et al.* studied the luminescence quenching of Ru(byp)_3_^2+^ as a means of monitoring oxygen in hexane at room temperature. Oxygen-sensitive tetraethoxysilane (TEOS), methyltriethoxysilane (MTMS) and tetramethoxysilane (TMOS)-based materials were prepared under different conditions. The materials were used in a flow-injection system and characterized in terms of quenching by oxygen dissolved in hexane, response time, and stability [101].

#### 2.2.3. Sol-gel materials

Sol-gel materials encompass a wide number of inorganic and organic/inorganic composite materials which share a common preparation strategy. The sol-gel process is a method for the synthesis of ceramic and glass materials at low temperature [102]. In this sense, Domínguez and co-workers have described a new full-field generic photonic biosensor approach, which relies on a bio-doped polymeric strip waveguide configuration. The authors showed the potential of tailor-made hybrid polymeric materials preparated by sol-gel technology for the fabrication of ultra-compact biosensor devices, where both the transducer and the recognition elements are merged into one single microstructure. The potential of this generic approach was demonstrated by developing a biosensor for the detection of H_2_O_2_ using horseradish peroxidase (HRP) as the doping agent. Moreover, the ease of fabrication the use of such polymeric materials are fully compatible with their integration in compact automatic analytical systems [103]. A new report presented organic-inorganic silicon-based polymers to obtain materials with mechanical, chemical, optical, or electrical properties. The controlled development of sol-gel microstructures using a simple fabrication technology may widen the range of promising applications of these materials in areas such as optoelectronics or chemical sensing [104].

#### 2.2.4. Organic ligands

Although not strictly analytical chemists, Costero’s group synthetized several chemosensor molecules that are very important in the field of fluorescent receptors. In this sense, several cyclohexyl-based fluorescent thioureas were synthesized for selective sensing of chiral dicarboxylates [105] such as TMA malonate in DMSO/water [106], TMA aspartate [107] and TMA maleate and fumarate [108]. An inhibition of FRET as a result of the complex geometry might be proposed as a transduction mechanism in the sensing process. Moreover, ligands trans-transoid-trans-1,2-bis(ethoxycarbonyl)-4,5-bis[3-(naphthalen-1-yl)thioureido]cyclohexane and trans-transoid-trans-1,2-bis(ethoxycarbonyl)-4-hydroxy-5-[3-(naphthalen-1-yl)thioureido] cyclohexane containing naphthalene units can be used as fluorescent sensors in the presence of appropriate dicarboxylates such as succinate and malonate [109]. This group synthesized and characterized several bipyridine derivatives such as (3,3′-bis(5-phenyl-1,4-dioxo-2,3,5-triaza)-2,2′-bipyridine) [110] and these ligands acted as chemosensor for α,ω-dicarboxylates that it was evaluated by UV-visible and fluorescence studies [111,112]. Finally, several biphenyl ligands have been prepared as fluorescent materials by this group. Homoditopic biphenyl thiourea derivatives have been prepared to be used in carboxylate sensing. Experimentals carried out with these ligands have demonstrated that the conformation of the free ligand has a strong influence on both complex stoichiometry and geometry [113,114]. Several bis-coronands from biphenyl have been prepared and their complexing and sensing properties for alkaline, alkaline-earth and transition cations have been studied [115] and one of them demonstrated to be a selective fluorescent sensor for mercury [116]. Several fluorescent macrocyclic ligands derived from biphenyl are described and these new compounds can be used in cation and anion recognition and sensing [117,118]. New ligands, derived from tetramethylbenzidine and containing additionally amino groups, are described. The behavior of the ligands were studied for sensing of different anions and cations [119]. Eight new polyazapodands containing a 4,4′-substituted biphenyl moiety have been synthesized. Four of them were functionalized on positions 4 and 4′ with a nitro group and other four with a dimethylamino substituent. The complexation properties of these ligands have been studied with Zn^2+^, Cd^2+^, Ni^2+^, Cu^2+^ and Pb^2+^, which show that the amino groups have a strong influence on the nature of the complexation and the fluorescent response of each ligand [120]. Díaz-García *et al.* synthesized 20 derivatives of 1,4,10,13-tetraoxa-7,16-diaza-cyclotadecane with fluorescence properties. The behavior of these materials has been studied into a flow-through cell in a FIA to determine alkali and alkali earth metal ions, overall for Mg^2+^ [121]. 17-member library of metallothionein-mimic decapeptides carrying a lariat ether group were synthesized. Each compound was screened, in the presence of europium (III) ions as fluorescent reporter for their sensing behavior towards metal ions (Cd^2+^, Hg^2+^, Cu^2+^, Mg^2+^ and Ca^2+^) using fluorimetric techniques [122].

#### 2.2.5. Other materials

Alegret *et al.* have reported a new electroactive material for potentiometric sensing of iodide and cyanide based on oil dispersion of AgI/Ag_2_S, which can be used for chemical and biological modifications [123]. C. Domínguez and co-workers have studied the application of Cl-ion sensitive ISFETs with photocured polyurethane-based polymer membranes with six different ionophores (ETH 9033, ETH 9009, MnTPPCl, organotin compounds in traditional ion-exchanger TDMACl) [124]. The group of M.D. Petit Domínguez designed and characterized a new organic-inorganic hybrid composite material to determine glucose, in presence of hydroxymethylferrocene as a redox mediator, in an electrochemical mode. This material is based on the entrapment of both gold nanoparticles (AuNPs) and glucose oxidase, which was chosen as a model, into a sol-gel matrix. The enhancement of the analitical response of the resulting biosensor induced by the presence of gold nanoparticles was better by comparison with a similar hybrid composite material without AuNPs [125]. E. Pinilla Gil *et al.* presented a simple procedure for the chemical synthesis of Bi nanoparticles and subsequent adsorption on screen-printed carbon electrodes offering reliable quantitation of trace Zn, Cd and Pb by anodic stripping square-wave voltammetry in nondeareated water samples [126]. A new material was fabricated by Díaz- García’s group based on porous chemical selective silicate particles for oxygen recognition in organic solvents. These materials operate on the principle of room-temperature phosphorescence quenching of a triplet probe which was entrapped in the silica network. A simple continuous flow system for oxygen sensing in a heptane/chloroform mixture was used [127]. In a new report, Díaz-García *et al*. have synthesized sorption material for pre-concentration of species, such as metal ions. It was reported the synthesis of different solid supports of anthracene-phosphine sulfide for Cu (II) and Pb (II) pre-concentration. Sensing properties of these materials were also evaluated using a flow-through optosensing approach [128].

## 3. Electrochemical Sensors

In the solid-state chemical (bio)sensor field, the oldest and the most widely used sensors have been the electrochemical sensors [129]. Electrochemical sensors, due to technical simplicity and fast responses, have revolutionized modern analysis and have received considerable attention due to their advantages, such as rapid and sensitive measurements, mass fabrication, low cost and decentralized in-field analysis with the possibility of miniaturization [7,130]. Charge transport between chemical phases or changes of electrical properties is detected due to chemical reactions on the electrochemical sensors [129]. This type of sensor can be classified as potentiometric, conductimetric, or voltametric sensors based upon their analytical principles of operation. Potentiometric sensors measure an equilibrium potential difference between a sensing electrode and a reference electrode; voltammetric sensors measure the current from the charge transport of an electrochemical reaction on a sensing electrode when a varying potential or a constant potential (amperometric detection) is applied between the working electrode and the solution sensors and conductimetric sensors quantitate the changes of electrical properties between two electrodes [129]. In this sense, Domínguez and co-workers have developed a novel design of an interdigitated electrode array (IDEA) impedimetric sensor with electrode digits separated by an insulating barrier. The transducer induces important changes in conductivity between the electrodes when the analytes link to the chemical modified surface [131]. This type of device has been used as a transducer to detect immunochemical and enzymatic reactions, as well as DNA hybridization events for direct label-free biosensor development. Three-dimensional sensor shows considerable improvement compared with a standard planar IDEA design [132]. Tuñón Blanco *et al.* presented the first label-free modified RNA-aptasensor for the detection of aminoglycoside neomycin B in whole milk. A competitive displacement assay was applied to the detection of aminoglycoside neomycin B using a fully 2′-O-methylated RNA aptamer with faradaic impedance spectroscopic (FIS) detection. Neomycin B in solution displaces the aptamer from its complex with the SAM-immobilized neomycin B. The reusable aptasensor is capable of discriminating neomycin B from paromomycin, which differs from it in the substitution of a single amine group with a hydroxyl one [133].

### 3.1. Electronic tongues

One of the most important sensor arrays with electrochemical detection are the electronic tongues. An electronic tongue can be defined as a group of arrays of chemical sensors in liquid samples together with its subsequent data processing [3]. The electronic tongue employs artificial neural networks (ANNs) as multivariate calibration algorithms to extract information from the cross-term responses [3]. The networks normally used are formed by three layers, an input layer, which takes the information from the sensor array, an intermediate layer that is responsible for learning, and an output layer, which provides the pursued chemical information. The number of neurons at the layers that exchange information is determined by the characteristics of the system being studied, whilst the number of neurons at the hidden layer has to be determined by trial and error. The available data are subdivided up to three subsets, namely the training, (internal) validation and test (or external validation), used for the quality-check and comparison of the obtained numerical models [3]. Domínguez *et al.* presented an electronic tongue device based on the multisensor ion selective field effect transistor (ISFET) array, sequential injection analysis (SIA) and partial least squares (PLS) method for data processing to offer an automation of the analysis of multicomponent liquids. Along with these, the system carried a custom-made flow cell for the sensor array and a cell for mixing liquid samples. The system was used for analyzing of the mineral water components (Na, K and chloride) [134]. Alegret *et al.* reported an electronic tongue, which determines nitrate ion in waters in the presence of chloride, one of its typical interfering anions [3]. In this sense, arrays of potentiometric sensors without selective response to specific analytes have been combined with the modeling abilities of the ANNs as an interesting approach for the simultaneous determination of analytes together with its interferents. The array is integrated by four potentiometric sensors, three nitrate ion-selective electrodes (ISEs) and a fourth chloride ISE. The indicator electrodes used were all-solid-state tubular flow-through electrodes, each one with a different PVC matrix membrane selective to the considered ions target [135]. Three different ion carriers for nitrate were used in order to induce a differentiated selectivity. The ionophores were tetraoctylammonium nitrate (TOAN), tridodecylmethylammonium nitrate (TDMAN) and tris(4,7-diphenyl-1,10-phenantroline) nickel(II) nitrate (TDPNN). The fourth sensor, intended to produce an independent measurement related only to chloride ion, employed the neutral carrier trioctyltin chloride (TOTC). The availability of the measuring system not requiring of any stage of interference removal would facilitate largely the development of specially robust and compact systems suited for the environmental monitoring of this parameter. The related automation aspects can be solved using the flow-injection analysis (FIA) technique. This sensor system once configured and optimised has been able to perform the direct determination of nitrate ion in complex samples. The signal was processing with a multivariate data treatment, in this case an artificial neural network based on the Bayesian regularization, allowed to quantify the concentration of nitrate between 0.1–100 mg L^−1^ without the need to eliminate chloride interference. Results obtained with this method were compared with the direct determination of nitrate using its ion-selective electrode, showing how the new strategy attains a better correlation of obtained versus expected values, especially at the lower concentration levels. The results demonstrated an interesting new way for the automated determination of species, aimed at the achievement of low maintenance monitoring systems, its long time applicability and its stability [3]. In other report, it was reported the design, construction, and applications of an electronic tongue based on an array of potentiometric sensors employing the Sequential Injection Analysis technique (SIA) operated as a Virtual Instrument implemented in LabVIEW6.1™. The new system has a serial of advantages, such as complete automation, easy handling, saving time, reliability and modularity [136]. This approach has been used in several ways during the last five years for S. Alegret and A. Mekoçi group using selective electrodes for different analytes. The principal purpose of this group is the use of advanced chemometrical tools in a simple way to obtain reduced information with an electronic tongue. The ion-selective polyvinyl chloride (PVC) membranes employed in the construction of the potentiometric sensor arrays were prepared by the authors. Table 2 shows a briefly summary of these articles.

### 3.2. Ion-selective electrodes

Electrochemical sensors, modified with different substrates, have been shortly reviewed. Alonso and co-workers proposed two analytical sensors based on ion-selective potentiometric sensor able to obtain *in situ* real-time measurements of the activity of the H^+^, Ca^2+^ and K^+^ ions in soils, respectively [150,151]. The potentiometric sensor system is based on potassium ion-selective electrodes. Sensors were built using PVC ion-selective membranes over an inner solid contact prepared with graphite-epoxy composites. A copper plate was used as a reference electrode. Three ion-selective sensors and three off-the-shelf temperature sensors and their associated circuits were mounted in a PVC tube to set up a soil probe [150]. For soil calcium and pH monitoring sensor system, the developed instrumentation was based on the connection of three solid-state ion-selective potentiometric sensors, three temperature sensors, and three moisture sensors at different heights. The pH was determined via potentiometry using a calcium chloride solution as the extractor and a combined glass electrode [151]. A two-stage electronic circuit composed of current and voltage amplifiers were designed to interface the sensors to a distributed data acquisition system and the data were transmitted via radio. The generated information allowed the monitoring of these parameters directly in soil, leading the possibility of making decisions in real time. In a new research line, the group of Ortuño has used a commercial ion-selective electrode body that permits the accommodation of a platinum counter electrode inside the inner filling solution compartment and, therefore, use of a four-electrode potentiostat with ohmic drop compensation. This device was used to apply two different double potential pulse techniques to detect ion transfers such as verapamil, clomipramine, tacrine, and imipramine [152,153].

### 3.3. Modified electrodes

Several groups have proposed different sensors based on modified electrodes. J.R. Castillo and co-workers studied the immobilization methods of tyrosinase enzyme (Tyr) on carbon-paste composite electrodes. Electrodes were based on the reversible inhibition of the enzyme and the chronocoulometric measurement of the charge due to the charge-transfer mediator 1,2-naphthoquinone-4-sulfonate (NQS). Tyr was immobilized onto electrodes using different procedures, such as entrapment within electropolymerized conducting and non-conducting polymers, covalent attachment to self-assembled monolayers (SAM), cross-linking with glutaraldehyde (and nafion covering) and dispersion within glassy-carbon (GC) electrodes. The analytical properties of the different biosensors were studied using dichlorvos organophosphate pesticide as the analyte. The best analytical properties were achieved using Tyr and NQS entrapment within an poly(*o*-phenylenediamine) polymer (*o*PPD) electropolymerized polymer, obtaining the GC-NQS-Tyr-PPD biosensor [154]. The group of J.M. Pingarrón has prepared several composite electrodes based on carbon paste, glassy carbon or graphite-Teflon electrodes to detect different compounds. The authors have developed colloidal gold-carbon paste electrodes (CPEs) by using CPEs modified with cysteamine (Cyst) to evaluate methionine solutions by recording cyclic voltammograms [155] and a xanthine oxidase (XOD) biosensor, based on a CPE modified with electrodeposited gold nanoparticles (nAu), to determine hypoxanthine (Hx) in sardines and chicken meat [42]. A tyrosinase biosensor was reported, based on the immobilization of the enzyme onto a glassy carbon electrode modified with electrodeposited gold nanoparticles (Tyr-nAu-GCE) to measure the bioelectrochemical polyphenols index in wines [156] and similar electrodes for improving amperometric detection of beta-galactosidase activity have been used to detect coliforms in drinking water [157]. Several graphite-Teflon composite electrodes have been developed by this group. A graphite-Teflon composite electrode matrix in which the enzyme and colloidal gold nanoparticles have been tested for catechol; phenol; 3,4-dimethylphenol; 4-chloro-3-methylphenol; 4-chlorophenol; 4-chloro-2-methylphenol; 3-methylphenol and 4-methylphenol in water samples [158]. Graphite-Teflon-glucose oxidase-peroxidase-ferrocene [159] and graphite-Teflon-peroxidase-ferrocene composite electrodes [160] were used to quantify bacterial pollution by monitoring glucose and hydrogen peroxide consumption, respectively. Other biosensor developed by this group was presented to determine L-lactic acid in yoghurt. The biosensor consists of an amperometric graphite-Teflon biosensor in which peroxidase (HRP), L-lactate oxidase (L-LOD) and the mediator ferrocene were immobilized [161]. Finally, a simple and new, third generation amperometric biosensor based on poly(vinyl)chloride tetrathiafulvalene-tetracyanoquinodimethane (PVC/TTF-TCNQ) composite electrode is proposed for glucose determination and glucose oxidase (GOx) was immobilized by crosslinking with glutaraldehyde. The tetrathiafulvalene-tetracyanoquinodimethane (TTF-TCNQ) salt acts as a conducting phase and as a redox mediator without needing the addition of any other substance [162]. For the first time, the group of E. Lorenzo have developed a *N*,*N’*-bis(dihydroxy-benzylidene)-1,2-diaminobenzene tetradentate ligands to modify glassy carbon (GC) electrodes giving rise to stable and redox active films. These modified electrodes present an electrochemical response strongly dependent on pH as can be anticipated for quinone/hydroquinone functional groups, and it has been applied to the construction of hydrazine sensors [163]. Electrodes modified with poly-[Ni^II^-DHS]/GC films showed a moderate electrocatalytic activity towards the oxidation of other aliphatic short chain alcohols, such as ethanol, 1-propanol, 2-propanol and n-butanol. In all cases, the catalytic currents presented linear dependences with the concentration of alcohol in alkaline solution [164]. In this sense, the authors modified glassy carbon electrodes with films of Prussian Blue [iron(III,II) hexacyanoferrate (II,III)]. The modified electrodes exhibited a reversible redox response, due to the oxidation/reduction of iron atoms present in the electrodeposited film. This sensor has been used as sensor for the determination of sulfite in several wine samples [165]. M.J. Arcos Martínez *et al.* presented a glassy carbon electrode modified with a polypyrrole (PPy) film, in which tyrosinase was immobilized. This enzymic amperometric electrode was used for carrying out chromium (III) measurements in spiked urine, waste water and river water samples based on the inhibitive action of this metal. [166]. M.D. Petit Domínguez *et al.* developed electrochemical sensors based on electrodes modified with entrapped ion-exchange polymers using the doping sol-gel method. Spectroscopic grade graphite electrodes were modified with the polymer-sol-gel solution using as anionic and cationic ion exchangers, such as poly(dimethyldiallylammonium chloride) (PDMDAAC) or poly(vinylsulfonic acid, Na salt) (PVSA). The polymer-sol-gel electrode surfaces were easily renewed by reversing the ion exchange reaction [167]. The determination of trace mercury species with positive charge has been carried with two of these modified electrodes: sol-gel and sol-gel-PVSA carbon composite electrodes and the developed electrodes showed very favorable electroanalytical properties for their use as amperometric sensors [168]. Two new electrodes consisting of sol-gel and sol-gel-poly(dimethyldiallylammonium chloride) (PDMDAAC) carbon composite electrodes have synthesized and characterized and both types of electrodes are capable of preconcentrating [Fe(CN)_6_]^4−^ from low-concentrated solutions [169].

Recently, screen-printed carbon electrodes (SPCEs) have been used for the development of several sensors and biosensors, due to the screen-printing microfabrication technology is nowadays well established for the production of thick-film electrochemical transducers [170]. Screen-printed electrodes are produced in a reproducible, inexpensive and mechanically robust way and show an important advantage such as the possibility of miniaturization. However, the functionalization of screen-printed electrodes is complicated and is difficult to control. Even so, the immobilization of antibodies or antigens on screen-printed electrodes has been carried out by physical or electrostatic adsorption [171], by sol-gel entrapment [172] or through the affinity reactions as biotin: streptavidin [173] or protein A or protein G [174], obtaining different immunosensor devices. During the last five years, Costa-García’s group has focused its work on the development of screen-printed immunosensors for several applications, which are summarized in Table 3.

J.M. Pingarrón’s group has developed a lectin-based screen-printed gold electrode to determine *Escherichia coli* using label-free transduction of the bacteria-lectin complex formation [189]. M.J. Arcos Martínez *et al.* used two electrodes, SPCEs and mercury coated SPCEs, to determine lamotrigine in pharmaceutical preparations [190] and antimony (III) in pharmaceutical preparations and seawater samples [191] by differential pulse adsorptive stripping voltammetry (DPAdSV). A new report presents SPCEs using as transducers for the peroxidase immobilization by pyrrole electropolymerization. The developed biosensor has been applied to the determination of levetiracetam (LEV) avoiding the pre-treatment of the samples [192]. This group reported a new enzymic electrochemical biosensor based on disposable SPCEs. Horseradish peroxidase was immobilized onto the carbon working electrode previously modified by an aryl diazonium salt. The formation of amide bonds between the amino and carboxylic groups of the enzyme surface, catalyzed by hydroxysuccinimide and carbodiimide, leads to the electrode functionalization. This biosensor was used to determine of LEV in complex matrixes such as pharmaceutical drugs [193]. Finally, M.J. Arcos Martínez *et al.* have described three-electrode configuration chips containing a Pt, Au and a screen-printed Ag/AgCl as counter, working and reference electrode, respectively. Selective determination of Phenobarbital (PB) in pharmaceutical drugs has been carried out by Cytochrome P 450 2B4 (CYP450) immobilization into a polypyrrole matrix onto the gold working electrode [194]. E. Pinilla Gil’s group has designed an electrochemical cell to allow fast, reproducible and highly efficient convective transport of dissolved substances to screen-printed electrochemical 3-electrode strips mounted on miniaturized plastic vessels. The experimental configuration was tested for Zn (II), Cd (II), and Pb (II), codeposited with Bi ions on a carbon disk screen-printed working electrode before detection by square wave anodic stripping voltammetry [195].

Using gold electrodes, E. Domínguez *et al.* described the use of multiple oligonucleotide sequences linked to an enzyme, glucose oxidase (GOx), for the detection of specific hybridization. An Au wire was used as working electrode which was treated with sodium salt of 3-mercapto-1-propane sulfonic acid. A redox layer as the cationic poly[(vinylpyridine)Os-(bpy)_2_Cl] redox polymer partially quaternized with bromoethylamine (RP), a catalytic layer as horseradish peroxidase (HRP_m_) and a RP layer were deposited. The transduction of the enzyme-linked DNA sensors is based on self-assembled multilayers, including a chemically modified anionic horseradish peroxidase electrochemically connected to a water-soluble cationic poly[(vinylpyridine) Os(bpy)2Cl] redox polymer in an electrostatic ordered assembly. Spectrophotometric characterization of these functionalized sequences results in an average number of three linked oligonucleotides per enzyme molecule and their specificity is demonstrated in both a direct and a sandwich-type hybridization assay. Hybridization is always performed at room temperature, and after 30 min of incubation, an the amperometric response obtained is proportional to DNA concentration [196].

E. Lorenzo and co-workers have described new genosensors. These sensors were carried out using gold electrodes modified with DNA via adsorption and [Os(bpy)2(phe-dione)]^3+/2+^ (bpy = 2,2′-bipyridyl) or [Os(phen)2(phen-dione)]^3+/2+^ (phen = 1,10-phenantroline) as electrochemical reported molecules. A thiol-linked single-stranded *Helicobacter pylori* DNA probe was immobilized, through S-Au bonds on to a gold electrode. Following hybridization with the complementary DNA sequence, the osmium complex was electrochemically accumulated within the double-stranded DNA layer. Electrochemical detection was performed by differential pulse voltammetry [197,198]. Gold electrodes modified by *N,N’-bis*(3,4-dihydroxybenzylidene)-1,2-diaminobenzene (3,4-DHS) and *N,N*’-bis(2,5-dihydroxybenzylidene)-1,2-diamino-benzene (2,5-DHS) as electrochemical probes in DNA sensing have also been developed. These ligands were capable of binding to double stranded DNA (ds-DNA) more efficiently than to single stranded DNA (ss-DNA). These biosensors have been constructed by immobilization of a thiolated capture probe sequence from *Helicobacter pylori* onto gold electrodes. After hybridization with the complementary target sequence, 3,4-DHS was accumulated within the double stranded DNA layer. Electrochemical detection was performed by differential pulse voltammetry over the potential range where the quinone moiety is redox active [199]. This group has also designed new bioanalytical platforms based on lactate oxidase (LOx) to carry out the analytical lactate determination. First of all, the LOx were immobilized by direct absorption on glassy carbon electrodes and highly ordered pyrolytic graphite and the LOx layers has been characterized using microscopic techniques. The lactate could be amperometrically determined [200]. In a new report, it was presented a lactate biosensor based on the immobilization of lactate oxidase (LOx) using two different strategies including direct adsorption and covalent binding onto gold surfaces. The characterization of the resulting lactate oxidase monolayers was performed using atomic force microscopy (AFM) and quartz crystal microbalance (QCM) techniques. In presence of lactate and using hydroxymethylferrocene as a redox mediator, biosensors obtained could be used as lactate biosensor in wine and beer samples [201]. E. Lorenzo’s group has constructed new biosensors to perform cholesterol determination, based on the covalently bonding cholesterol oxidase (ChOx) to a 3,3′-dithiodipropionic acid di(N-succinimidyl ester) (DTSP)-modified gold electrode [202] or the direct adsorption on gold electrodes [203]. Exhaustive characterizations of both the immobilization processes and the morphological properties of the resulting ChOx monolayer were performed *via* a quartz crystal microbalance (QCM) and atomic force microscopy (AFM). Tuñón Blanco and co-workers have reported a polymerase chain reaction (PCR) assay targeting the 16S-rRNA gene of *L. pneumophila* giving rise to a 95-mer amplicon. Amplicons were hybridized to a biotin-labeled reporter sequence and then to a thiolated stem-loop structure immobilized onto gold electrodes as a reporter molecule with 1-naphthyl phosphate as a substrate. 1-Naphthol enzymically generated was determined by differential pulse voltammetry (DPV) [204].

M.J. Arcos Martínez and co-workers have development a β-cyclodextrin (β-CD)-based sensor to determine rifampicin (RIF). β-CD was fixed onto a Pt electrode by pyrrole electropolymerization and RIF was deposited on the surface of the modified electrode through complex formation and quantified amperometrically. This sensor was applied to the RIF determination in pharmaceutical preparations and biologic samples [205]. J.R. Castillo *et al.* have constructed three cholesterol biosensors based on the formation of a layer of Prussian-Blue (PB) on a Pt electrode for the electrocatalytic detection of the H_2_O_2_ generated during the enzymic reaction of cholesterol with cholesterol oxidase (ChOx). The enzyme was entrapped within a polypyrrole (PPy) layer electropolymerizated onto the PB film. The influence of the formation of SAMs on the Pt surface on the adherence and stability of the PB layer and the formation of an outer layer of nafion (Nf) as a means of improving selectivity were both studied [206]. Another cholesterol biosensor was designed based on the enzyme cholesterol oxidase (ChOx) and subsequent reconstitution of the apo-protein with a complexed flavin adenine dinucleotide (FAD) monolayer. The charge transfer mediator pyrroquinoline quinone (PQQ) was covalently bound to a cystamine SAM on an Au electrode. The effective release of the FAD from the enzyme and the successful reconstitution were verified using molecular fluorescence and cyclic voltammetry [207].

Tuñón Blanco and co-workers have reported an electrochemical genosensor for the detection of nucleic acid sequences specific of *Legionella pneumophila*. An immobilized thiolated hairpin probe is combined with a sandwich-type hybridization assay, using biotin as a tracer in the signaling probe, and streptavidin-alkaline phosphatase as reporter molecule. The activity of the immobilized enzyme was voltammetrically determined by measuring the amount of 1-naphthol generated after two min of enzymic dephosphorylation of 1-naphthyl phosphate. The sensor allows discrimination between *L. pneumophila* and *L. longbeachae* with high sensitivity under identical assay conditions (no changes in stringency). Experimental results show the superior sensitivity and selectivity of the hairpin-based assay when compared with analogous sandwich-type assays using linear capture probes [208]. This hairpin-DNA probe was compared with a new stem-loop DNA probes (SPE) with the same geometry and desing. A lower quantification limit is obtained with SPE and in addition, the selectivity is improved [209]. A new article described the potential of those nucleic acids probes whose molecular recognition ability relies on a conformational change (e.g., folding/unfolding mechanism) in electrochemical sensing. It provides an overview of the toolbox of assays using these probes for genosensors and aptasensors, highlighting its performance characteristics and the prospects and challenges for biosensor design [210].

### 3.4. Electrochemical flow-through sensors

Domínguez *et al.* constructed a multianalyte flow electrochemical cell (MAFEC) incorporating amperometric enzyme carbon paste electrodes for simultaneous carbohydrate analysis. The cell operates as a radial flow thin-layer device and can achieved mass transport controlled response for fast electrochemical reactions. All the enzymatic sensors are mediated with different osmium compounds appropriate for each enzyme’s mechanism (NAD or PQQ dehydrogenases), combining in some cases, multienzyme sensors. The integrated system was used for the simultaneous bioelectrocatalytic detection of fructose, sucrose, glucose, galactose, and lactose in food samples, such as juice and milk samples with good results [211]. J.A. Ortuño *et al.* have developed new solvent polymeric membrane ion sensors incorporated in a flow-injection system. These amperometric sensors were based on a plasticized poly(vinyl) chloride (PVC) membrane. The flow-through cell incorporated the four electrodes and the membrane, which contained tetrabutylammonium tetraphenylborate. The determination of tetrabutylammonium was studied and two different amperometric methods, indirect and direct, were also developed for the determination of dodecylsulfate [212], tracine [213], tiapride [214], sulpiride [215] and catamphiphilic drugs such as the antiarrhythmic drugs procainamide and quinidine, the antimalarial quinine and the anesthetics bupivacaine, lidocaine, procaine and tetracaine [216]. Finally, a solvent polymeric membrane ion sensor has been applied to study the ion transfer of several ionic liquid cations, from water to a poly(vinyl chloride) membrane plasticized with 2-nitrophenyl octyl ether and the study has mainly been focused on dialkylimidazolium and alkylpyridinium cations [217].

## 4. Optical Sensors

Optical transducers are based on optical phenomena detecting the intensity of photon radiation that reaches a sensor, which are the result of an interaction of analyte with receptor [129,5]. Different optical transduction techniques have been applied in several sensors, such as absorbance, reflectance, fluorescence, phosphorescence, chemi-/bioluminescence, refractive index, surface-plasmon resonance (SPR), optothermal effect and light scattering [4,5]. Due to its facility of solving, a wide variety of analytical problems, optical chemical sensors have been used in different fields, including industry, environment, and clinical analysis [4].

Fluorescence- and phosphorescence-based sensors require bulky excitation sources, so they are not as compatible with miniaturized solid-state photosensors and electronics [129]. Molecular absorption spectroscopy is no doubt the most frequently used detection technique in analytical laboratories due to its high flexibility for adaptation to a wide variety of analytical problems. In general, these sensors are comprised of various reagents immobilised within suitable membranes. A number of the existing optodes utilises of color complexion reactions between immobilized ligands and heavy metal ions [4]. There are two basic measurement methodologies using liquid-phase chemiluminescence (CL) reactions: static samples and flowings streams. Flowing stream methods involve delivery and mixing of the CL reagent with the analyte stream or column effluent and the use of a flow-cell for the detection of the CL emission at a fixed time after mixing. FIA includes relatively simple methodology offering acceptable robustness, feasibility and precision, and its rapid measuring response makes it suitable for monitoring liquid-phase CL reactions. This technique allows the sample to undergo on-line chemical and physical treatment to obtain species suitable for CL detection [4]. L.F. Capitán-Vallvey *et al.* have worked in the development of a serial of disposable optical sensors changing the ionophores and chromophores in the sensing film and using the optical transduction technique according to the applications. A summary is presented below. Capitán-Vallvey *et al.* prepared one-shot biosensors for lactate determination [218–220]. In one case, the electrochemiluminescence system was based on a Methocel™ membrane placed on the graphite working electrode of the screen-printed electrochemical cell. The lactate recognition system was based on the generation of hydrogen peroxide by lactate oxidase (LOx) and the subsequent reaction with electrochemically oxidized luminol. The measurements of the electrochemiluminescence were made by conventional photomultiplier detectors. The biosensor was tested for the analysis of lactate in human saliva [218]. The second sensor was based on chemiluminescence measurements and the lactate recognition system LOx. The transduction system consisted of luminol, peroxidase from *Arthromyces ramosus* (ARP) and metallic aluminum, all immobilized in a polyion complex membrane. The proposed method used conventional chemiluminescence instrumentation and the composition of the membrane and reaction conditions were optimized for obtaining adequate sensitivity. The performance of the chemiluminescent biosensor was tested for the analysis of lactate in yoghurt [219]. Finally, the last one-shot sensor was based on screen-printed electrodes. The lactate recognition system was based on lactate oxidase and the transduction system consisted of electro-oxidation of luminol, with all the reagents immobilized in a Methocel™ membrane. The luminometer was based on a large silicon photodiode as detector [220]. The same authors have studied and characterized the reactivity of six different ionophores double-armed crown ethers based on an 18 atoms ring against alkaline and alkaline-earth ions set on a plasticized PVC membrane. The ionophores are incorporated in optical membranes working on ion-exchange. The use of the same chromoionophore as transducer permits to extract conclusions on the influence of lipophilicity and size of the terminal group of the side chain on calcium selectivity. The ionophore that contains an (N-adamantylcarbamoyl) acetyl moiety originated was the most selective membrane for calcium. Analytical parameters for calcium determination using prepared membranes were calculated [221]. LCapitán-Vallvey *et al.* presented a serial of disposable sensors based on ion-exchange mechanism for potassium determination. The one-shot sensor used in these papers is a potassium sensor, previously developed and characterized by the Capitán-Vallvey group [222], based on lipophilized Nilo Blue as the chromoionophore changing its color from violet to blue when the potassium concentration increases. Several detection techniques were used to acquire the analytical parameters, such as a conventional flatbed scanner [223], transflectance and chromaticity systems [224], and transmitted intensity with a solid state photodetector due to color change in the sensing film [225] for quantitative determination of potassium. Potassium sensing films were produced on a polyester substrate with PVC, 2-nitrophenyloctyl ether (NPOE), dibenzo-18-crown-6-ether (DB18C6), the chromoionophore N,N-diethyl-5-(octadecanoylimino)-5H-benzo[a]phenoxazine-9-amine (ETH 5294) and potassium tetrakis (4-chlorophenyl) borate (TCPB). The sensing area of the disposable sensor is a red 12-mm-o.d. circular film with a calculated thickness around 4.5 *μ*m. Spectroradiometric measurements of reflectance and CIELAB hue-angle were carried out. The trueness of both procedures was demonstrated comparing it with results obtained by a DAD spectrophotometer used as a reference measurement procedure. The usefulness of the procedure was checked by determining K(I) in different types of waters and beverages [226]. Moreover, Capitán-Vallvey and co-wokers have presented a disposable multisensor based on ionophore-chromoionophore chemistry for optical monitoring of potassium, magnesium and hardness in water. The selectivity for each species comes from the different ionophore included in every membrane, but the optical transduction uses the same chromoionophore liphophilised Nile Blue. The analytical procedure uses a black and white non-cooled Charge Coupled Device (CCD) camera for image acquisition of the one-shot multisensor after reaction, followed by data treatment for quantitation using the effective absorbance as analytical parameter. The trueness of this multisensor procedure was demonstrated comparing it with results obtained by a DAD spectrophotometer used as a reference. Finally, it was satisfactorily applied to the analysis of these analytes in miscellaneous samples, such as water and beverage samples from different origins [227]. A study of different oxygen sensitive membranes taking into account membrane polymer, luminophore and stabiliser concentrations has been performed for oxygen response and photobleaching behavior [228]. Besides, Capitán-Vallvey *et al.* developed an easy-to-use portable instrument based on a microcontroller for atmospheric oxygen concentration measurement. Optical sensors based on luminescence quenching have thoroughly investigated in the search for sufficient sensitivity and high stability. The microcontroller-based instrument uses a LED as optical excitation and a binary output photodetector, coated with the oxygen sensing film, for collecting the luminescent emission. The dye was synthesized with platinum octaethylporphyrin complex immobilised in a polystyrene membrane. This membrane selecting as an oxygen probe was stabilised with the heterocyclic amine DABCO [229]. It was reported a calibration procedure that compensates for the temperature dependence in a gaseous oxygen measurement system. The proposed calibration function has been shown to be applicable for different sensing film thicknesses and luminophore concentrations using the same fittings parameter. Additionally, this function has successfully applied to other oxygen dyes. Good agreement has also found when the performance of the instrument was compared to a commercially available portable instrument based on an electrochemical sensor [230]. In a new report, Capitán-Vallvey *et al.* researched a new optical disposable sensor to determine mercury. The optical sensor has a circular sensing zone containing all the reagents necessary to produce a selective response to mercury and it was formed by plasticised PVC incorporating the cation-selective neutral ionophore 1,4,7,10-tetraazacyclododecane, the chromoionophore 9-dimethylamino-5-[4-(15-butyl-1,13-dioxo-2,14-dioxanonadecyl)phenylimino]benzo [a] phenoxazine and tetrabutyl-ammonium tetraphenylborate as lipophilic salt. The measurement principle is based on an ion-exchange mechanism. The sensor was introduced for a period of time depending on the mercury content into a water sample at a pH of 4.7 and a color change from blue to red took place, allowing the photometric measurement. The procedure was applied to the determination of mercury in different types of waters (tap, mineral and spring), validating the results against a reference procedure [231]. The sensor used a polyester sheet with a sensing area composed of plasticized PVC that incorporates a tetraarylborate salt as Hg (II) selective recognition reagent and a porphyrin proton-selective fluoroionophore as the optical transducer. The sensing scheme is based on the decomposition of tetraarylborate anion induced by Hg(II) taken by the membrane which compels the deprotonation of the porphyrin, recovering its fluorescence. The fluorescence increases after 15 min contact with Hg (II) at pH 2.4. The disposable sensor presents good selectivity to Hg (II) over other metal ions [232]. L.F. Capitán-Vallvey and co-workers have designed a portable radiometer and it was used to carry out nitrate measurements with a disposable ionophore-chromoionophore sensor. A light emitting diode (LED), with a dominant wavelength of 660 nm, was used as the illumination source. The one-shot sensors are used directly without any prior conditioning and the absorbance is measured with the portable radiometer before and after equilibration with the sample. The performance of the optical one-shot sensor was tested for the analysis of nitrate in different types of natural water (tap, river, well and sea) [233]. New optical biosensors based on the intrinsic absorption properties of peroxidase have been proposed by J. Galban *et al.* During the reversible reaction between peroxidase (HRP) and H_2_O_2_, several peroxidase intermediate species, showing different molecular absorption spectra, are formed, which can be used for H_2_O_2_ determination. The designed biosensor consists of HRP and glucose oxidase entrapment in a polyacrylamide gel matrix and it has been used to determine glucose in fruit juices, synthetic serum samples and blood without sample pretreatment [234,235]. This biosensor based on glucose oxidase (GOx) entrapped polyacrylamide (PAA) film was placed in a flow cell to determine glucose in blood in an automatic mode [236]. Finally, a biosensor prepared by HRP entrapment in a polyacrylamide gel matrix has been applied in hydrogen peroxide and peracetic acid determinations in wastewaters [237]. C. Domínguez and co-workers have developed the first absorbance biosensor based on pure silicon hollow integrated waveguides. The use of horseradish peroxidase (HRP) as a model recognition element, an enzymatic sensor for the measurement of hydrogen peroxide was fabricated and experimentally characterized. The simple technology proposed in this work enables the fabrication of cost-effective, easy-to-use, miniaturized biosensor generic platforms, these being envisioned as excellent candidates for the development of lab-on-a-chip systems [238].

### 4.1. Optical fibers

Fiber-optic sensors are the most common type of optical sensors, but they are not suitable for complementary metal-oxide-semiconductor CMOS integration (even with on-chip waveguides) because the indirect band gap of silicon makes it difficult to generate the light source [4,239]. Application of many of the optical phenomena in sensors became possible through the using optical fibers in various configurations [9]. Many of the implemented systems are based on direct spectroscopies that range from UV to IR, and from absorbance to fluorescence and surface plasmon resonance [240].

A polymer optical fiber (POF) is a type of optical fiber that is used to guide the excitation light from the source to a small sensing area, where the fluorescent selective membrane is deposited and another POF is used to collect the light back to the electronics where it is processed [1]. In the last years, the use of POFs in optochemical sensing applications is increasing, mainly in the field of evanescent sensors, fluorescent sensors and in medical applications. There are a great variety of POF sensors depending on the characteristic POF employed. The significant advantages of POF sensors are large core size, large numerical aperture (NA), flexibility, easy handling, ruggedness and lower cost [1]. Castillo *et al.* have developed a sensor film of polyaniline for sulfite [241] and vitamin C [242] determination in wine, in pharmaceutical preparations, and commercial fruit juices, respectively. Chemical polymerization of aniline was carried out and a very thin film of polyaniline was obtained. This sensor is based on the changes in absorption properties because of the redox interaction with polyaniline. The higher absorbance variation was observed at 550 nm and 700 nm for sulfite and vitamin C, respectively. J. Alonso *et al.* fabricated a novel fluorescence dip-probe POF sensor that allows performing *in situ* chemical determinations such as phosphate (PO_4_^3−^) [243] and lead determination in soil samples [1]. The lead sensor was chemically activated with a lead-selective plasticized PVC (polyvinylchloride) membrane containing a new synthesized suitable fluoroionophore and a commercial lead ionophore [1]. The results indicate good response of the sensors and the potential applicability of these probes is to assist in the monitoring of soil nutrients. Optical sensors based on organic conducting polymers have thus become a promising solution [244]. These polymers have optical properties in the VIS-near IR region being compatible with light-emitting diodes (LEDs) and diode lasers, which are the most frequently used excitation sources in optical sensors. They combine the advantages of optical sensors (long distance measurement and freedom from electrical interference being the most important), with those of conducting polymers (the polymer itself acts as both indicator and support so that no leaching into the solution is observed) [245]. Focusing on the development of a miniaturized fluorescence sensor, two research lines have been followed in the group of J. Alonso. The first one deals with the development of miniaturized instrumentation for *in situ* fluorescent measurements and the second one, the formulation and characterization of fluorescent optode membranes, compatible with the previous one. In this way, integrated waveguide absorbance optodes (IWAOs) are miniaturized optical transduction platforms, which confer excellent analytical properties to bulk optodes, such as high sensitivity, reduced response times and versatility, depending on the ion-selective membrane composition and confer robustness, mass production capabilities and high sensitivity. However, the design, formulation and integration of the membranes are still key factors for modulating sensitivity, selectivity, response time and dynamic range. A proper selection of the main compounds involved in the recognition process is essential [11,246]. In the last years, miniaturized fluorescent optodes have been developed as an attractive alternative to absorbance optodes. A typical fluorescent optode consists of a plasticized PVC matrix, which contains an ionophore selective to the analyte, a proton-selective fluoroionophore and, a lipophilic ionic salt to maintain the electroneutrality within the membrane and to promote the ionic exchange between the bulk and the solution in contact. Fluoroionophores, as the key elements in such optodes, is responsible of the optical signal and it should present a high fluorescence quantum yield, good solubility in the plasticized PVC matrix, high lipophilicity and high photostability [247]. Under the first research line, J. Alonso *et al.* reported a novel compact dual-wavelength measurement system based on digital lock-in amplification. It was designed for optical absorbance sensors and it has been evaluated using an integrated waveguide absorbance optode (IWAO), which was previously developed in the research group. This device consisted of a chemically active polymeric membrane deposited over an optical integrated planar waveguide circuit and offered the advantages of integrated optics (miniaturization of the sensing system and reduction of costs and size) and those of chemically active membranes (increase of sensitivity of the sensor while reducing the response time). The system has been successfully tested with some of these IWAOs, showing an adequate correction of undesired effects such as membrane hydration [248]. Besides, it was set out to synthesise different cyanine compounds for their use as pH chromoionophores in bulk optodes [249–251]. Most of them were included in bulk optodes, demonstrating their potential for their use as chemical sensors for absorbance [252,253] and fluorescence measurements [254], and some others were also applied in IWAOs [255], giving promising results. In this way, new far-visible absorbing hexamethine–hemicyanine [247,255], nortricarbocyanine [11], norcyanine [256], ketocyanine [257] and heptamethine cyanine [253] dyes have been synthesised for future application as chromoionophores in IWAOs based on bulk optodes or a miniaturized optical fluorosensor. The sensor has sucessfully characterized showing good analytical features. Sanz Medel’s group described the electronic design and the performance of a low-cost fiber-optic instrument for pH fluorescent measurements. The chemical sensing phase consists of an organic pH indicator (mercurochrome) immobilized in a sol-gel matrix placed at the end of a fiber optic by means of a steel grid. The active phase was excited by means of a high-intensity blue light-emitting diode and fluorescence emission is detected by a low-cost photodiode. To perform such measurements, two fiber-optic measurement channels were used. The sensor is useful over the pH range of 4–8, providing highly reliable results [258,259].

### 4.2. Optical flow injetion sensors

A sensitive flow-through immunosensor has been used for mycotoxin zearalenone in cereal samples by Moreno-Bondi’s group. The sensor was based on a direct competitive immunosorbent assay using a horseradish-peroxidase-labeled derivatized for the binding sites of a rabbit polyclonal antibody and fluorescent detection [260]. Moreover, the development of a flow-through solid-phase room temperature phosphorescence (RTP) sensor based on the energy transfer from a phosphor molecule to an orthophosphate dye-indicator has described by Sanz-Medel’s group. This sensor was applied to determine of orthophosphate in aqueous samples. After injection, the phosphomolybdenum blue is on-line co-immobilized onto a polymeric resin containing adsorbed erythrosine B. This selected donor molecule exhibits strong RTP in a de-oxygenated aqueous media when retained on the surface of polymeric resin beads [261]. This sensor has applied for the rapid identification of aflatoxin belonging to *Aspergillus genus* [262]. M.E. Díaz García *et al.* have presented a study of the analytical application of a nafcillin-imprinted sol-gel to the direct determination of the β-lactam antibiotic in spiked milk-based samples using a room temperature phosphorescent flow-through system. The influence of the sample matrix on the transduction and the recognition processes were statistically determined, and results demonstrated that the imprinted sol-gel optosensing system could be effectively applied to real sample analysis [263]. J.A. Ortuño *et al.* have described a flow-through spectrophotometric bulk optode for the flow-injection determination of thiocyanate in human saliva. As active constituents, the optode incorporates the lipophilized pH indicator 5-octadecanoyloxy-2-(4-nitrophenylazo)phenol and methyltridodecyl ammonium chloride, dissolved in a plasticized poly(vinyl)chloride membrane entrapped in a cellulose support [264]. A. Fernández-Gutiérrez’s group developed several flow-through phosphorescence optosensors for several analytes. An optical sensor based on a non-ionic resin (Amberlite XAD-4) solid support in a continuous-flow system was used to detect and quantify the highly carcinogenic polycyclic aromatic hydrocarbon (PAH) benzo[a]pyrene (BaP) [265] and to characterize 15 PAHs such as naphthalene, acenaphthylene, acenaphthene, fluorene, phenanthrene, anthracene, fluoranthene, pyrene, chrysene, benzo(a)anthracene, benzo(k)fluoranthene, benzo(b)fluoranthene, benzo(a)pyrene, indeno(1,2,3-cd)pyrene, benzo(g,h,i)perylene and dibenzo(a,h)anthracene) [266] in water samples. Then, a photomultiplier device with an artificial neural network as transducer based on a nonionic resin (Amberlite XAD-4) solid support in a continuous flow system has been used for screening of four polycyclic aromatic hydrocarbons: anthracene(ANT), benzo[a]pyrene (BaP), fluoranthene (FLT), and benzo[b]fluoranthene (Bbf). The optosensor proposed was satisfactorily applied to the determination of the considered PAHs in water samples in presence of the other 12 EPA-PAHs [267]. New papers present the development of a single flow-through phosphorescence optosensor based on a nonionic resin solid support (Amberlite XAD 7) for determination of pesticide N-1-naphthylphthlamic acid (NAP) [268] and simultaneous determination of NAP and its metabolite 1-naphthylamine (NNA) [269]. This group describes two luminescence methods based on a flow-through optical sensor to determine 2-naphthoxyacetic acid. A fluorescence optosensor based on the online immobilization of 2-naphthoxyacetic acid (β-NOA) on a non-ionic resin (Amberlite XAD-7) solid support in a continuous-flow system and a phosphorescence optosensor based on the online immobilization of β-NOA on silica gel solid support were developed and compared [270]. Maquieira *et al.* have developed immunosensors for detecting atrazine en extra virgin oil [271], herbicide glyphosate in water and soil samples [272], and several pollutans such as carbaryl, 1-naphthol and irgarol 1051 [273]. In all the cases, fluorescence detection was used and all of them were very selective showing no cross-reactivity to other similar compounds to corresponding analyte. The same group presented a rapid immunosensor based on the homogeneous competition among the analyte, a fluorescent tracer, and the antibody, followed by separation of free and bound species by means of a restricted access alkyl-diol silica C_18_ reversed-phase chromatografic support. The sensor capabilities are demonstrated through the analysis of natural waters [274]. In addition, this group has been proposed a isocyanate-ended poly(methyl methacrylate) (PMMA)-modified Digital Discs as a support to perform DNA hybridization assays. Measurements were carried out with a CD player by detecting an enzymic reaction [275]. An analytical application in microarray format of a cancer marker (alpha-fetoprotein, AFP) and a selective herbicide (atrazine) on four types of audio-video disk was conducted [276]. Numerous flow-through fluorescence-based optosensors using to detect several analytes have been developed by the group of A. Molina-Díaz and a summary was presented in Table 4.

### 4.3. Surface plasmon resonance

Optical transmission through minuscule structures gained a new impulse with the advent of the surface plasmon resonance (SPR) technique, by directing light waves to the interface between a metal and a dielectric [301]. SPR presents several advantages such as high-speed, high sensitivity, label-free method for biomolecule detection and detects biochemical binding without any labeling process.

Lechuga *et al.* developed a new fully automated biosensor based on a label-free immunoassay format intended for the real-time analysis of several compounds. This immunosensor is based on a portable β-SPR device commercialized by Sensia, S.L. (Spain), which works under reversible conditions, allowing the continuous monitoring of a high number of samples. The size and electronic configuration of the device allow its portability and utilization on real contaminated locations. The immunoassay was based on a binding inhibition test by using the covalent immobilization of an analyte derivative onto the gold-coated sensing surface. An alkanethiol self-assembled monolayer (SAM) was formed on the gold-coated sensor surface in order to obtain a reusable sensing surface. L.M. Lechuga’s group, in collaboration with D. Barceló’s group has been used this system to determine atrazine, [302], organochlorine (DDT) and its metabolites [303], carbaryl [304], chlorpyrifos [305–307] in natural water samples, 3,5,6-trichloro-2-2-pyridinol (TPC), a metabolite of chlorpyrifos in urine samples [308] and human growth hormone (hGH) in human serum samples [309]. Multi-analyte detection of environmentally relevant pesticides was carried out by the commercial SPR device with a special design. Two-chanelled SPR biosensor allows the determination of several analytes (DDT, chlorpyrifos and carbaryl) in a multi-analyte way [310]. Different applications of SPR have been carried out by group of L.M. Lechuga. It has synthesised a copolymer between chitosan and anionic polyacrylic derivatives, bearing sulfonic moieties, where occurred the protein. The complexation process allows a real time monitoring of different surface molecular interactions with very high sensitivity. The acrylic macromolecules are two families of copolymers of 2-acrylamido-2-methylpropane sulfonic acid (AMPS) and, respectively, 2-hydroxyethylmethacrylate (HEMA) and N,N’ -dimethylacrylamide (DMAA). SPR can be used for a simple “*in vitro*” protein adsorption analysis, by flowing aqueous solutions of albumin and fibrinogen. While both proteins were adsorbed on the uncomplexed chitosan, the complexed coatings exhibited different and very promising behaviors. In particular, they showed no adsorption or only selective adsorption of albumin [311]. Besides, the authors have studied a novel magneto-optic surface-plasmon-resonance (MOSPR) sensor for biomolecules detection. This physical transduction principle is based on the combination of the magneto-optic activity of magnetic materials and a SPR of metallic layers. Such a combination can produce a sharp enhancement of the magneto-optic effects that strongly depends on the optical properties of the surrounding medium, allowing its use for biosensing applications. Optimization of the metallic layers and the experimental setup could result in an improvement of the limit of detection by as much as 1 order of magnitude [312]. Metal films perforated by nanoholes constitute a powerful platform for surface SPR biosensing. It was found that the refractive index sensitivity of nanohole arrays increases if their resonance is red-shifted by increasing the separation distance between holes. However, an additional sensitivity enhancement occurs if the nanohole sensors are manufactured on low index substrates, despite the fact such substrates significantly blue-shift the resonance [313].

### 4.4. Mach-Zehnder interferometric (MZI) biosensor

Mach–Zehnder interferometric (MZI) biosensor is one of the most interesting, due to their high sensitivity and the possibility of optoelectronic integration in lab-on-a-chip microsystems [314]. The Mach-Zehnder interferometer (MZI) uses an optical waveguides as the basic element of their structure for light propagation and is based on the evanescent wave field sensing. The optical waveguides have two main features: monomode behavior and a high surface sensitivity. To obtain both features, L.M. Lechuga *et al.* have designed and fabricated a total internal reflection (TIR) nanodevice on silicon technology and a MZI microdevice based on arrow waveguide. The design and fabrication have been described in detail and direct biosensing with both sensors has been tested after a specific receptor coupling to the surface device using nanometer scale immobilization techniques [315]. Then, fabrication, characterization and packaging of novel multilevel microfluidic-optical waveguide biosensor arrays have been successfully fabricated. The integrated device consists of a three-dimensional embedded microchannel network fabricated using enhanced CMOS compatible SU-8 multilevel polymer technology on top of a wafer containing MZI nanophotonic biosensor devices. PMMA housing provides connection to the macro-world and ensures robust leakage-free flow operation of the devices. The devices have been designed to operate under continuous flow [315,316]. A real-time detection of the covalent immobilization and hybridization of DNA strands [316] and detection of single nucleotide polymorphisms at BRCA-1 gene, involved in breast cancer development, without target labeling have been carried out [317].

### 4.5. Attenuated total reflection Fourier sensors

Several straightforward membrane-based sensors, which use attenuated total reflection Fourier transform infrared (ATR-FT-IR) spectroscopy have developed. The flow cell designed permits the on-line micro-liquid-liquid extraction of the target analyte into an organic solvent layer (OSL), which was deposited on the ATR surface using a sequential injection manifold. The aqueous and organic phases were separated via a commercial hydrophobic membrane placed on the polytetrafluoroethylene (PTFE) piece of the cell. Finally, the analytical performance of the design was established for the detection and quantitation of Triton X100 in water [318], surfactant and oil total indices in industrial degreasing baths [319] and caffeine in soft drinks [320].

### 4.6. Slot-waveguide based refractometric sensors

Refractive index (RI) sensors based on slot-waveguide microring resonators have been recently proposed. They were fabricated in two different material systems Si_3_N_4_ and SiO_2_ by A. Maquieira’s group [321]. In the slot-waveguide based refractometric sensors a change in RI of the probed region causes a corresponding phase shift that can be detected as either a frequency or an intensity shift when the guided optical probe interacts with the sample to be tested. For applications requiring the analysis of a liquid sample and the sensing signal can be employed to determine the RI of the sample as compared to a reference sample. Binding events on the sensor surface are monitored through the measurement of resonant wavelength shifts with varying biomolecular concentrations.

### 4.7. Cantilever sensors

Cantilever-based sensors are very attractive transducers for physical, chemical and biological sensors due to simplicity, wide range of sensing domains [322] and extremely high-sensitivity when cantilever is scaled-down into sub-micrometer scale dimensions [323]. Microcantilevers deflect or deform owing to changes in binding or surface stress following a specific molecular recognition event. Recent developments combine the latest IC and CMOS technologies to produce intelligent cantilevers [324], extremely small cantilevers [325], or large arrays [326]. In general there are three different methods to transducer the recognition event into a micromechanical motion: first, the frequency change due to additional mass loading or a change in the force constant can be measured (*i.e.*, the cantilever is used as a microbalance); second the bending of a bimetallic cantilever can be used as temperature sensor (e.g., to sense calorimetric effects upon adsorption); additionally, cantilevers can work as stress sensors by measuring the bending due to changes in the surface stress at one side of the cantilever [327]. Optical read-out is one of the most common schemes for detecting the movement of microcantilevers. The displacement of the free end of the cantilever is measured using the optical deflection of an incident laser beam on a position-sensitive photodetector, which allows the absolute value of the cantilever displacement to be calculated [328]. Deflection of a microcantilever caused by any kind of biochemical reaction occurring on its surface can be detected with subangstrom resolution if an appropriate detection technique is exploited [329]. L.M. Lechuga and co-workers have worked to design and fabricate cantilever arrays aimed to develop an integrated biosensor microsystem. Arrays of up to 33 microcantilevers are fabricated based on spin coating in the novel polymer material SU-8. The mechanical properties of SU-8 cantilevers, such as spring constant, resonant frequency and quality factor are characterized as a function of the dimensions and the medium. The devices have been tested for measurement of the adsorption of single stranded DNA [330–332]. In the field of the applications of microcantilever sensors, L.M. Lechuga *et al.* have studied the interaction forces responsible for the bending motion during the formation of a self-assembled monolayer of thiolated 27-mer single-stranded DNA on the gold-coated side and the subsequent hybridization with the complementary nucleic acid. Detection of nucleic acid hybridization with nanomechanical sensors requires reference cantilevers to remove nonspecific signals, more sensitive microcantilever geometries, and immobilization chemistries specially addressed to enhance the surface stress variations [333].

### 4.8. Commercial optical biosensors

The RIver ANAlyser (RIANA) is a multi-analyte immunosensor based on a rapid solid-phase indirect inhibition fluoroimmunoassay that takes place at an optical transducer chip chemically modified with an analyte derivative. The transducer surface is chemically modified with the analyte derivative placed in different discrete locations. Fluorescence produced by labelled antibodies bound to the transducer is detected by photodiodes and it can be correlated with the analyte concentration. The sensor surface can be regenerated thus allowing the performance of several measurements (around 300) with the same transducer [332]. The group of D. Barceló has worked on the use of this immunosensor for determine several chemical such as bisphenol A [333] and simultaneous multi-analyte determination of estrone, isoproturon and atrazine [334,335] in natural waters.

## 5. Piezoelectric Sensors

### 5.1. Quartz crystal microbalance

Mass-sensitive sensors detect the change of mass on a sensing layer. In the case of chemical sensors, the mass changes arise from absorption, evaporation, deposition, or erosion due to chemical reactions. Several sensing structures have been employed to detect these mass changes, such as the thickness shear mode (TSM) resonator, quartz crystal microbalance (QCM), and surface acoustic wave (SAW) device. Most of these devices are not suitable for integration with CMOS circuits [336]. The mass-based sensors that can incorporate CMOS circuits use the resonant frequency shift of a cantilever beam to detect the change in mass [337,338].

Mass-sensitive sensors transform the mass change at a specially modified surface into a change of property of the support material. The mass change is caused by accumulation of the analyte. In this category can be grouped piezoelectric devices and surface-acoustic-wave devices. The vibration of piezoelectric crystals produces an oscillating electric field in which the resonant frequency of the crystal depends on its chemical nature, size, shape and mass. By placing the crystal in an oscillating circuit, the frequency can be measured as a function of the mass. When the change in mass (m) is very small compared to the total mass of the crystal, the change in frequency (f) relates to m, as follows: AF = Cf^2^Am/A, where f is the vibration frequency of the crystal in the circuit, A is the area of the electrode and C is a constant determined in part by the crystal material and thickness. Piezoelectric crystals, sometimes referred to as a quartz-crystal microbalances (QCMs), are typically made of quartz and operate at frequencies between 1–10 MHz. These devices can operated in liquids with a frequency –determination limit of 0.1 Hz, the limit of detection (LOD) of mass bound to the electrode surface is about 10^−10^–10^−11^ g. Limitations for this transduction method involve format and calibration requirements [5].

The group of A. Ríos and M. Valcárcel have worked using piezoelectric sensors, such as quartz crystal microbalance (QCM) in flow injection systems. The quartz crystal microbalance has been used to determine sulphur dioxide in wines with a mercury- amalgamated surface [339], carbonate in soil samples without functionalized surface [340], caffeine in coffee and tea samples [341] and vanillin in food samples [342] by using a supported liquid membrane sensor with MIP. J.R. Castillo *et al.* have tested a piezoelectric immunosensor for ochratoxin A (OTA) detection. In this case, OTA–bovine serum albumin (OTA–BSA) conjugate was immobilized on gold surface quartz crystal. Three different immobilization procedures such as direct adsorption and covalent attachment to two alkane-thiol self-assembled monolayers for OTA-BSA were studied. Covalent attachment of the OTA–BSA conjugates through gold was also tested obtaining a higher signal [343].

### 5.2. Cantilever

A. Ríos and co-workers have presented a piezoelectric microcantilever-based CO_2_ gas sensor measuring frequency shifts. The detection scheme makes use of the dependence of the resonance frequencies on the gas pressure. The performance of the microcantilever was better than the QCM sensor. This microcantilever sensor was used, afterwards, for developing and validating an analytical method for carbonate determination in soil samples based on the selective generation of carbon dioxide with hydrochloric acid [344].

## 6. Commercial Spanish Devices

Several companies from analytical research groups such as Sensia, Dropsens and Inbea Biosensors are working to develop and commercialize analytical instrumentation for life sciences laboratories and environmental measurements.

SENSIA develops and commercializes portable multibiosensor systems, based on technologies developed by Biosensor Group within the National Microelectronics Centre (CNM) of the Spanish National Research Council (CSIC). This company commercializes a Sensia β-SPR Research Platform. SENSIA β-SPR is based on the Kretschmann configuration and it is used to achieve the resonant condition by total internal reflection. The instrument consists of a polarised laser diode light strikes into two flow cells, with a volume of 300 nl, a prism and a multi-photodiode that are located on concentric rotary stages with an angular resolution of 0.01. β-SPR system has a detection limit of 10^−5^ in refractive index. Replaceable sensor glass slides are 10 mm x 10 mm coated with a 50 nm Au layer. The surface chemistry functionality is achieved by self assembled monolayers or thin films. Monitoring changes in refractive index as a function of time allows real-time analysis of the binding events occurring at the sensor surface due to the continuous flow of buffers and samples provided by the flow injection system. SENSIA β-SPR can be applied in several fields such as chemical and biochemical sensing, drug discovery, diagnostics, proteomics, genomics, forensics, food analysis and environmental monitoring [345].

DropSens is an Innovative Technology-Based Firm founded with the aim of being a leader in the electrochemical biosensors market and its activity is located in Oviedo (Asturias), Spain. DropSens develops and commercializes biosensors based on thick-film hybrid technology. These biosensors, in combination with portable instrumentation, use microvolumes of sample and are applied in different areas such as clinical analyses or environmental control. Several devices have been commercialized such as several potentiostats that can be applied for voltammetric, amperometric or potentiometric measurements, screen-printed carbon electrodes as working and counter electrodes, bismuth oxide modified screen-printed carbon electrodes to determine heavy metals at ppb levels, multi- and single- walled carbon nanotubes modified screen-printed electrodes that show the electrocatalytic properties, screen-printed carbon electrodes modified with co-phthalocyanine, Meldola’s Blue, Prussian Blue, or ferrocyanide to develop enzymatic biosensors, screen-printed gold electrodes and screen-printed platinum electrodes used as working electrodes. Moreover, DropSens has the technology to develop screen-printed electrodes based on customer’s designs. Several flow-cells for screen-printed electrodes have too commercialized [346]. Several Spanish groups have been used these electrodes to develop different sensors such as the group of J.M. Pingarrón [80,188,190,347–349], P. Tuñón-Blanco [211], E. Pinilla Gil [196] and A. Costa-García [185,187,350].

Inbea Biosensores S.L. is a spin-off company of the Complutense University of Madrid and it was founded in 2007 by a group of Doctors from the Department of Analytical Chemistry. Inbea provides analytical instrumentation to the food and agriculture market and developes and sells easy to use, fast, reliable, portable and low cost analytical instrumentation based on amperometric enzymatic biosensors for determining different parameters. The Inbea analytical instrumentation system consists of a Bioanalyzer system and different types of biosensors, depending on the analyte that is going to be measured. The amperometric enzymatic biosensors are devices comprising of a biological recognition element (enzyme or enzymes), in contact with an electrochemical transducer. The enzymes selectively detect the substance being analysed, producing a biochemical signal. This biosensor is capable of detecting glucose, fructose, ethanol, lactic and malic acid and gluconic acid. The Bioanalyzer system consists of a potentiostat, a sensor unit, two measuring cells, one reference electrode and three reagent vials and comes in both single-channel and bi-channel models, in order to be able to perform measurements and controls on two analytes at the same time [351].

## 7. Conclusions

This review presents some of the representative works in the development of new sensors by Spanish analytical groups. The development of sensors is of interest to researchers in the field of analytical chemistry because sensors provide a simple and rapid alternative for the detection of several compounds. Other important advantages of sensors are the possibility of portability and miniaturization leading the development of lab-on-a-chip and carrying out analysis in real time. Recent advances in the technological field and the use of new materials with molecular recognition properties, such as carbon nanotubes, micro and nanoparticles, magnetic beads, MIPs, polymers and metal complexes, have led sensor modifications to increase in selectivity, a marked sensitivity and simplification of the analytical devices. This article shows an overview of the various types of sensors, the transducers used for sensor development, the design of experimental devices and the applications are summarized. The contribution of the Spanish research groups can be considered as very relevant, both for basic developments and for applied and technical uses.

## Figures and Tables

**Table 1.** Spanish analytical groups and developed sensors.

**Investigation group**	**Responsible**	**Developed Sensors**	**Links**
Sensors and biosensors group (University Autonoma Barcelona)	Salvador Alegret	- Electrochemical sensors (synthesis of electrochemical compounds, genosensors, electronic tongues	http://webs2002.uab.es/_c_gr_gsb/index.html
Sensors and biosensors group (University Autonoma Barcelona)	Julián Alonso	- Potenciometric sensor devices (ion selective electrodes) - Optical sensor: polymer optical fiber	http://einstein.uab.es/_c_gr_gsb/GSB/julian/index.htm
Electroanalysis group (University of Pais Vasco)	Ramón J. Barrio	- Polimeric microelectrodes	--
Electroanalysis group (University of Burgos)	María J. Arcos-Domínguez	- Sensitive and selective electrodes modified with nanomaterials, enzymes, polymers and organic compounds	http://www.ubu.es/paginas/grupos_investigacion/cien_biotec/elan/index.htm
Environmental chemistry group (IDAEA-CSIC, Barcelona)	Damiá Barceló	- Optical sensor: SPR and RIver ANAlyser (RIANA) applications	http://grac.org/Barcelo.pdf
Analytical spectroscopy & sensors group (University of Zaragoza)	Juan R. Castillo	- Optical sensors (based on organic conducting polymers)	http://www.unizar.es/departamentos/quimica_analitica/personal/jr_castillo.html
Solid-phase spectrometry group (University of Granada)	Luis F. Capitán-Vallvey	- Optical sensors: disposable optosensors	http://www.ugr.es/~efasesol/inicial.htm
Inmunoeletroanalysis group (University of Oviedo)	Agustín Costa	- Electrochemical sensors: no-enzymatic, enzymatic sensors and genosensors (screen-printed carbon electrodes)	http://inmunoweb.iespana.es/proyectos.htm
Organic chemistry of metal complexes group (University of Valencia)	Ana M. Costero	- Optical sensors (based on fluorogenic and cromogenic species)	http://www.uv.es/supraorg
Optical sensors and bioanalysis group (University of Oviedo)	Marta E. Díaz-García	- Optical sensors: phosphorescence and fluorescence sensors	http://www.uniovi.es/vicinves/web_vieja/portal/ot/activos/075.pdf
Microelectronic institute of Barcelona	Carlos Domínguez	- Nanofabrication and functional properties of nanostructures - Transducers for chemical and biochemical sensing	http://www.imb-cnm.csic.es/index.php?lang=en
Bioelectrochemistry and biosensors group (University of Alcalá)	Elena Domínguez	- Electrochemical sensors (inmunoassays)	http://www.uah.es/INVESTIGACION/INVESTIGACION/docs/Grupos/CC_Experimentales.pdf
Techniques and methods of chemical analysis group (UNED)	Jesús S. Durand	- Luminescent sensors (based on molecular imprinted polymers)	http://www.uned.es/gtymaq/1.htm
Environmental, biochemical and foodstuffs analytical control research group (University of Granada)	Alberto Fernández-Gutiérrez	- Optical sensors (based on metalic oxides and molecular imprinted polymers)	http://feugr.ugr.es/ProyectoConecta/php/contenidogrupo.php3?id=5
Analytical spectroscopy & sensors group (University of Zaragoza)	Javier Galban	- Biosensors (based on organic conducting polymers)	http://www.unizar.es/departamentos/quimica_analitica/JavierGalban.html
Nano biosensors and molecular biophysics group (Nanoscience & Nanotechnology Investigation Centre, CSIC, Barcelona)	Laura M. Lechuga	- Optical sensor: SPR, microcantilevers	http://www.cin2.es/biosensores
Biosensors group (University of Autonoma of Madrid)	M. Encarnación Lorenzo	- Amperometric (bio)sensors (composite electrodes)	http://www.uam.es/gruposinv/biosens/presentacion.html
Institute of applied molecular chemistry (University of Valencia)	A. Maquieira	- Chemicals sensors (optical and electrochemical (bio)sensors), new materials and microelectronic equipments	http://iqma.webs.upv.es/eng/index.php
Nanobioelectronics & biosensors group (Catalonian Institut of Nanotecnology, Barcelona)	Arben Merkoçi	- Electrochemical (bio)sensors modified with nanostructurated materials	http://nanocat.uab.cat/dataeng/recerca/biopriv/bio_home.php
Analytical chemistry group (University of Jaen)	Antonio Molina	- Optical sensors: phosphorescence and fluorescence sensors	http://www.ujaen.es/serv/vicinv/verGrupo.php?grupo=42
Laboratory of optical sensors (University Complutense of Madrid)	María C. Moreno-Bondi	- Fluorescent optical sensors: luminiscent (bio)sensors array	http://www.ucm.es/info/gsolfa
Automatic analytical method and chemical sensors group (University of Murcia)	Joaquín A. Ortuño	- Optical and piezoelectric sensors	https://curie.um.es/curie/catalogo-grupo-investigacion.du?cods=E044*02
Chemical sensors and biosensors (University of Autonoma of Madrid)	María. D. Petit-Domínguez	- Electrochemical and optical (bio)sensors	http://www.uam.es/gruposinv/biosens
Electroanalysis and electrochemical (bio)sensors group. (University of Complutense of Madrid)	José M. Pingarrón	- Electrochemical sensors (composite, screen-printed and gold electrodes)	http://www.ucm.es/info/analitic
Chemical analysis of environmental (University of Extremadura)	Eduardo Pinilla-Gil	- Electroanalytical methods and sensors for heavy metals in environmental samples.	http://www.unex.es/quianaelec/aquima
Automatization, simplification and miniaturization of analytical processes (University of Castilla La Mancha)	Ángel Ríos	- Piezoelectric sensors (QCM and microcantilever)	http://www.uclm.es/organos/vic_investigacion/GruposUCLM/grupos.aspx?gr=191&inf=per
Chemometrics, qualimetrics and nanosensors group (Universitat Rovira i Virgili)	Xavier Rius	- Electrochemical sensors (FET electrodes)	http://www.quimica.urv.es/quimio/ang/maincat.html
Analytical spectrometry (University of Oviedo)	Alfredo Sanz-Medel	- Optical sensors: phosphorescence and fluorescence sensors	www12.uniovi.es/spectrometry
Electroanalysis group (University of Oviedo)	Paulino Tuñon-Blanco	- Electrochemical genosensors, modified electrodes and piezoelectric sensors	http://www.uniovi.es/electroanalisis/englishversion.htm
Automation, simplification, miniaturization and quality of the (bio)chemical measurement processes (University of Cordoba)	Miguel Válcarcel	- Piezoelectric sensors	http://www.uco.es/grupos/FQM-215/index.htm

**Table 2.** Selective electronic tongue developments.

**System**	**Indicator electrode**	**No. of electrodes**	**Detection technique**	**Analyte**	**Sample**	**Detection mode**	**Reference**
e-tongue, ANN and FI	Ion-selective PVC polymer membrane	4	Potentiometric sensor	Nitrate, Chloride	Water	Direct detection	[3]
e-tongue, ANN and FI	Ion-selective PVC polymer membrane	8	Potentiometric sensor	NH_4_^+^, K^+^ and Na^+^ ions.	Synthetic and river water, waste water and fertilizer	Simultaneous multi-determination	[137]
e-tongue, ANN and SIA	Home-made epoxy-graphite electrode	--	Voltammetric sensor	o-cresol, pchlorophenol, 4-chloro-3-methylphenol	--	Direct and simultaneous multi-determination	[138]
e-tongue, ANN and SIA	Ion-selective PVC polymer membrane	5	Potentiometric sensor	Cl^−^, NO_3_^−^ and HCO_3_^−^	Water	Direct and simultaneous multi-determination	[139–141]
e-tongue, ANN and FI	Ion-selective PVC polymer membrane	--	Potentiometric sensor	--	Commercial waters, orange-based drinks, tea samples and natural juice	Direct detection	[142]
e-tongue, ANN	--	--	Voltammetric sensor	Tryptophan, cysteine, and tyrosine	Animal “feed”	Direct detection	[143,144]
e-tongue, ANN	Ion-selective PVC polymer membrane	8	Potentiometric sensor	Ammonium, potassium, sodium, chloride, phosphate and nitrate ions	Soils	Direct and simultaneous multide-termination	[145]
Bioe-tongue and ANN	Urease and creatinine deiminase covalently immobilized onto ammonium selective electrodes and polymeric membranes	--	--	Urea, creatitine, ammonium, potassium and sodium.	Clinical samples	Direct and simultaneous multi-determination	[146]
e-tongue, ANN	Screen printed on polymeric substrate	5	Potentiometric sensor	Ammonium, potassium, sodium, chloride and nitrate ions	Surface waters	Direct and simultaneous multi-determination	[147,148]
e-tongue, ANN and SIA	Two based on chalcogenide glasses Cd and Cu sensor, and the rest on PVC membranes Pb and Zn sensor.	4	Potentiometric sensor	Cd, Cu, Pb and Zn	--	Direct and simultaneous multi-determination	[149]

**Table 3.** Selective screen-printed immunosensors.

**Indicator electrode**	**Functionalization**			**Detection technique**	**Analyte**	**Reference**
	**Type of immobilization**	**Enzimatic label**	**Substrate**			
SPCEs	Streptavidin/Biotin reaction	Alkaline phosphatase (AP)	3-indoxyl phosphate (3-IP)	Voltametric sensor	Virulence nucleic acid in Pneumolysin and autolysin genes of the human pathogen *Streptococcus pneumoniae*	[175]
SPCEs and flow system	--	Alkaline phosphatase (AP) and Horseradish peroxidase (HRP)	3-indoxyl phosphate (3-IP)	Voltametric sensor	--	[176]
SPCEs	Streptavidin/Biotin reaction	Platinum (II) complex	--	Voltametric sensor	Virulence nucleic acid in Pneumolysin and autolysin genes of the human pathogen *Streptococcus pneumoniae*	[177]
SPCEs	Streptavidin/Biotin reaction	Alkaline phosphatase (AP)	3-indoxyl phosphate (3-IP)	Voltametric sensor	Rabbit IgG in direct determination Competitive immunoassay	[170]
Comercial SPCEs Flow cell	Horseradish peroxidase (HRP)	--	3,3′,5,5′-Tetramethylbenzidine (TMB)	Amperometric sensor	Interleukin 6	[178]
SPCEs Flow cell	Alkaline phosphatase	--	P-Nitrophenyl phosphate	Amperometric sensor	p-nitrophenol	[179]
Glassy carbon electrodes	--	Gold complex	--	Voltametric sensor	SARS virus	[180]
--	Streptavidin/Biotin reaction	Alkaline phosphatase (AP)	3-indoxyl phosphate (3-IP)	Voltametric sensor	DNA	[130,181]
Carbon SPCEs	Streptavidin/Biotin reaction	Alkaline phosphatase (AP)	3-indoxyl phosphate (3-IP) and silver ions	Voltametric sensor	Virulence nucleic acid in autolysin gene of the human pathogen *Streptococcus pneumoniae*	[182]
Gold SPCEs	--	--	Polycarbonate and alumina	Voltametric sensor	Potassium ferricyanide, p-aminophenol, indigo carmine, silver nitrate and ferrocene	[183]
SPCEs	p-aminophenol- phosphatase	Alkaline phosphatase (AP)	MWCNT-COOH	Voltametric sensor	p-aminophenol	[184]
SPCEs	--	Gold nanoparticles	--	Voltametric sensor	Lead	[185]
SPCEs	--	Alkaline phosphatase (AP) and gold nanoparticles	3-indoxyl phosphate (3-IP)	Voltametric sensor	SARS (severe acute respiratory syndrome) virus	[186]
SPCEs carbon, gold or carbon nanotubes	--	Alkaline phosphatase (AP)	3-indoxyl phosphate (3-IP)	Voltammetric sensor	Prostate specific antigen (fPSA and tPSA)	[187]
Gold nanostructured SPCEs	--	--	--	Voltammetric sensor	Lead in blood	[188]

**Table 4.** Selective flow-through fluorescence-based optosensors.

**System**	**Detection technique**	**Active sorbent substrate**	**Reagents**	**Analyte**	**Reference**
Bead injection spectroscopy-flow injection analysis (BIS-FIA)	Spectrofluorometry	Sephadex QAE A-25	Morin (2′,3,4′,5,7-pentahydroxyflavone)	Berillium and Aluminum	[277]
Flow injection (FI) manifold	Spectrofluorometry	Sephadex SP C-25 cation-exchange gel beads	--	Diphenhydramine in pharmaceutical samples	[278]
Flow injection (FI) manifold	UV spectrophotometry	Sephadex SP G-15 sorption gel	--	Ciprofloxacin	[279]
Flow injection (FI) manifold	Spectrofluorometry		--	Quinine (QN) and quinidine (QD)	[280]
Bead injection spectroscopy-flow injection analysis (BIS-FIA)	Spectrophotometry	Sephadex QAE A-25 resin	Ferrozine (FeFz_3_) ^4−^	Promethazine and trifluoperazine in pharmaceutical samples	[281]
Bead injection spectroscopy-flow injection analysis (BIS-FIA)	Spectrophotometry	Sephadex QAE A-25 resin	Prussian blue (PB)	Ascorbic acid	[282]
Flow injection (FI) manifold	UV spectrophotometry	Octadecyl silane C_18_ gel	--	Methylxanthines: caffeine (CF) and theophylline (TP) in pharmaceuticals samples and CF and theobromine (TB) in food and beverages.	[283]
Flow injection (FI) manifold	Spectrofluorometry	C_18_ silica gel	--	Pesticides: carbendazim (CBZ), carbofuran (CF) and benomyl (BNM)	[284]
Bead injection spectroscopy-flow injection analysis (BIS-FIA)	Spectrofluorometry	Sephadex QAE A-25 resin	1,2-dihydroxyanthrquinone-3-sulfonic acid (Alazarin Red S)	Vanadium (V)	[285]
Flow injection (FI) manifold and multioptosensors	UV spectrophotometry		--	Salicylamide (SLC) and caffeine (CF)	[286]
Flow injection (FI) manifold	Spectrofluorometry	Sephadex SPC-25 microbeads	--	Furosemide and triamterene in human urine and blood serum.	[287]
Flow injection (FI) manifold	Fourier transform (FT) Raman spectroscopy	Sephadex QAE A-25 resin	--	Sulfonamides: sulfathiazole and sulfamethoxazole	[288]
Flow injection (FI) manifold	Luminescence technique	--	A luminiscent TB chelate	Pipemidic acid and quinolone antibacterial agents (norfloxacin, ciprofloxacin, enoxacin, trovafloxacin) in biological fluids	[289]
Bead injection spectroscopy-flow injection analysis (BIS-FIA)	UV spectrophotometry	Sephadex QAE A-25 anion exchange gel	2-carboxyl-2-hydroxy-5-sulfoformazylbenzene (Zincon)	Biparametric mixtures (copper (II) and zinc (II))	[290]
Flow injection (FI) manifold	Chemiluminiscency	--	--	Salicylic acid	[291]
Flow injection (FI) manifold	UV spectrophotometry	--	--	Ternary pharmaceutical mixture	[292]
Flow injection (FI) manifold	UV spectrophotometry	C_18_ silica gel	--	Flufenamic acid (FFA)	[293]
Flow injection (FI) manifold	UV spectrophotometry	C_18_ silica gel	--	Azoxystrobin residues in grapes, musas and wines	[294]
Flow injection (FI) manifold	Spectrofluorometry	C_18_ silica gel	--	Imidacloprid in peppers and environmental waters	[295]
Flow injection (FI) manifold	Spectrofluorometry	C_18_ silica gel	--	Pesticides: a-naphthol, o-phenylphenol and thiabendazole in water samples	[296]
Flow injection (FI) manifold	Spectrofluorometry	C_18_ silica gel	--	Naproxen and salicylic acid in biological samples	[297]
Flow injection (FI) manifold	Spectrofluorometry	C_18_ silica gel	p-(tosylamino) quinoline	Zinc (II) in drinking water	[298]
Flow injection (FI) manifold	Luminescency	Sephadex QAE A-25 anion exchange gel	Tb (III) and lanthanide-sensitized luminiscence	p-aminobenzoic acid (PABA)	[299]
Sequential injection análisis (SIA)	Spectrofluorometry			Vitamins B2, B6 and C	[300]
